# Design, synthesis and biological activity of *N*^4^-phenylsubstituted-7*H*-pyrrolo[2,3-*d*]pyrimidin-4-amines as dual inhibitors of aurora kinase A and epidermal growth factor receptor kinase

**DOI:** 10.1080/14756366.2017.1376666

**Published:** 2017-11-08

**Authors:** Sonali Kurup, Bradley McAllister, Pavlina Liskova, Trusha Mistry, Anthony Fanizza, Dan Stanford, Jolanta Slawska, Ulrich Keller, Alexander Hoellein

**Affiliations:** a College of Pharmacy, Roosevelt University, Schaumburg, IL, USA;; b Department of Chemistry, Harper College, Palatine, IL, USA;; c III. Medical Department, Technische Universität München, Munich, Germany;; d German Cancer Consortium (DKTK) and German Cancer Research Center (DKFZ), Heidelberg, Germany

**Keywords:** Pyrrolo[2,3-*d*]pyrimidines, aurora kinase inhibitors, epidermal growth factor receptor kinase inhibitors

## Abstract

Simultaneous inhibition of multiple kinases has been suggested to provide synergistic effects on inhibition of tumour growth and resistance. This study describes the design, synthesis and evaluation of 18 compounds incorporating a pyrrolo[2,3-*d*]pyrimidine scaffold for dual inhibition of epidermal growth factor receptor kinase (EGFR) and aurora kinase A (AURKA). Compounds **1**–**18** of this study demonstrate nanomolar inhibition of EGFR and micromolar inhibition of AURKA. Compounds **1**–**18** allow for a structure–activity relationships (SAR) analysis of the 4-anilino moiety for dual EGFR and AURKA inhibition. Compound **6**, a 4-methoxyphenylpyrrolo[2,3-*d*]pyrimidin-4-amine, demonstrates single-digit micromolar inhibition of both AURKA and EGFR and provides evidence of a single molecule with dual activity against EGFR and AURKA. Compound **2**, the most potent inhibitor of EGFR and AURKA from this series, has been further evaluated in four different squamous cell head and neck cancer cell lines for downstream effects resulting from AURKA and EGFR inhibition.

## Introduction

Dysfunctional epidermal growth factor receptor kinase (EGFR) plays a role in tumour progression and angiogenesis in squamous cell carcinoma of the head and neck (SCCHN), non-small cell lung cancer (NSCLC) and colorectal cancer^1^^,^^2^. Initial therapeutic strategies for EGFR inhibition have focused on selective EGFR inhibitors including the monoclonal antibody, cetuximab and small molecule kinase inhibitors, erlotinib and gefitinib. Irreversible EGFR inhibitors, afatinib and osimertinib have been approved for use more recently^3^. However, tumours have redundant signaling pathways for tumour progression and often develop resistance to single EGFR inhibitors^3^. One of the mechanisms of resistance to cetuximab in SCCHN has been redundant signaling mediated by aurora kinases^4^^,^^5^. Aurora kinase A (AURKA) is a serine/threonine kinase that involved in regulating mitotic entry, centrosome maturation and separation. Hoellein et al.^6^ demonstrated that resistance to cetuximab in SCCHN can be overcome when given in combination with the AURKA inhibitor, alisertib. Astsaturov et al.^7^ reported that EGFR inhibitors and AURKA inhibitors synergised to reduce cell viability and tumour size. Chen et al.^8^ showed that AURKA upregulation played a role in gefitinib sensitivity in NSCLC cells and suggested that AURKA and EGFR inhibitors given in combination could be effective. Zhang et al.^9^ also reported that combination therapy with erlotinib and alisertib had synergistic effects in lung cancer cell lines *in vitro* and *in vivo*. Single agents with multi-targeted inhibitory attributes have been successful in the treatment of cancer^10–14^. Dual inhibition of EGFR and AURKA could offer synergistic mechanisms to overcome resistance and suppress tumours such as SCCHN and NSCLC where redundancy in EGFR and AURKA signaling is observed^6–9^. Thus, it was of interest to determine if a single molecule could be developed for dual AURKA and EGFR inhibition based on the crosstalk between EGFR and AURKA and the development of resistance to single EGFR inhibitors.

**Figure 1.**
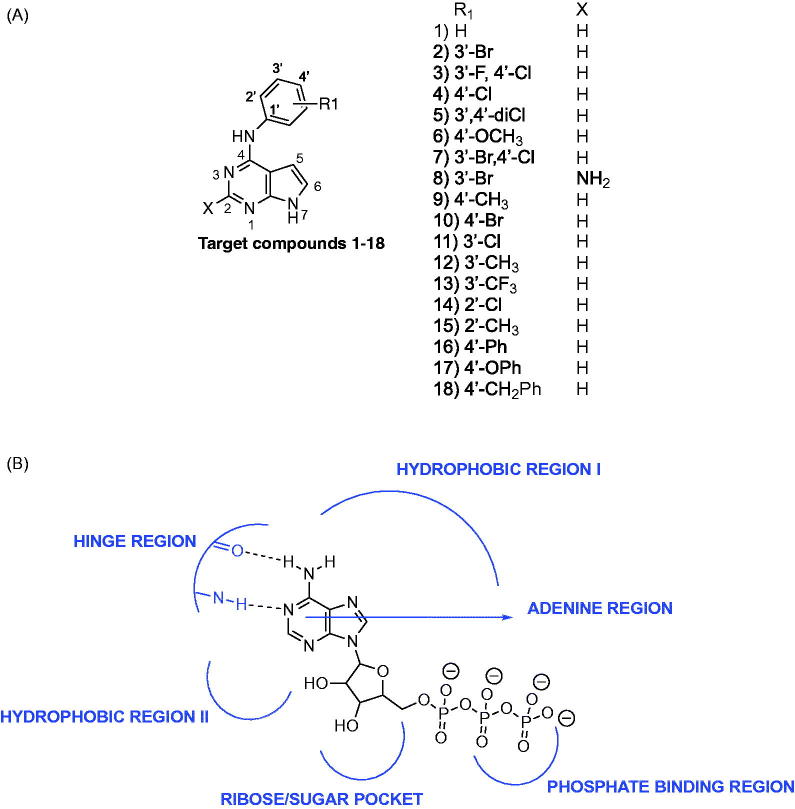
Target compounds **1–18** tested for dual activity against AURKA and EGFR.

The pyrrolo[2,3-*d*]pyrimidine heterocycle is a privileged scaffold that has been optimally substituted for varied kinase inhibition^15–19^. Varied 4,6-disubstituted and 4,5,7-trisubstituted pyrrolo[2,3-*d*]pyrimidines have been described as EGFR inhibitors as part of patents^15^^,^^16^. Gangjee et al.^13^^,^^17^ previously reported 4,6-disubstituted pyrrolo[2,3-*d*]pyrimidines and 4,7-disubstituted pyrrolo[2,3-*d*]pyrimidines as multi-targeted inhibitors of EGFR, platelet derived growth factor receptor kinase β (PDGFRβ) and vascular endothelial growth factor receptor kinase (VEGFR). Cheng et al.^18^ developed 2,5,7-trisubstituted pyrrolo[2,3-*d*]pyrimidines as irreversible EGFR inhibitors. Le Brazidec et al.^19^ reported 2,4,7-trisubstituted pyrrolo[2,3-*d*]pyrimidines as selective aurora kinase inhibitors; however, the compounds were not evaluated against EGFR. Thus, it was of interest to determine if the pyrrolo[2,3-*d*]pyrimidine scaffold could be appropriately substituted to incorporate dual AURKA and EGFR inhibition. To this end, compounds **1**–**18** (Figure 1) incorporating a minimally functionalised pyrrolo[2,3-*d*]pyrimidine scaffold were developed to investigate potential dual EGFR and AURKA inhibition.

The ATP binding site of kinases is a well-defined pocket comprising of six different regions^1^. ATP binds to the hinge region, adenine region and sugar pocket. Hydrophobic region I which is found in the back cleft of the ATP pocket is not occupied by ATP and has been explored in many kinase inhibitors to improve potency and selectivity. Electron withdrawing groups (Cl, Br, CF_3_) and electron donating groups (CH_3_, OCH_3_) were incorporated at varied positions in the 4-anilino moiety of pyrrolo[2,3-*d*]pyrimidines (Figure 1). Two substitutions (3′-Br, 3′-F, 4′-Cl) that were previously successful for EGFR inhibition when incorporated in pyrrolo[2,3-*d*]pyrimidines and other heterocyclic scaffolds were utilised in compounds **2** and **3** respectively to determine impact on dual EGFR and AURKA inhibition^14^. Additional substitutions with different electronic and steric properties were also incorporated in the 4-anilino moiety and docked within EGFR and AURKA.

Molecular modeling studies on compounds **1**–**18** within the ATP pocket of EGFR were conducted using a reported pyrimido[4,5*-b*]azepine-EGFR complex (PDB code: 3W33) as the starting point (Figure 2, panel A)^20^. One hydrogen bond to the hinge region and interactions with the adenine region of the ATP binding site was defined as the key pharmacophore for binding to EGFR. The pyrrolo[2,3-*d*]pyrimidine ring in **1**–**18** occupied the adenine region with two hydrogen bonds with the hinge region. A hydrogen bond was observed between the *N*^1^ of the pyrimidine in **1**–**18** and the backbone NH of Met793, and a second hydrogen bond was observed for the pyrrole NH hydrogen and the backbone carbonyl of Met793. An additional dipole-dipole bond was observed between C-2 and the backbone carbonyl of Gln791 in the hinge region. The 4-anilino moiety (R_1_) of **1**–**18** occupied a hydrophobic pocket in the back cleft of the ATP site and was found to superpose on the anilino moiety observed in the reported EGFR inhibitor, pyrimido[4,5-*b*]azepine. Molecular modeling studies for compounds **1**–**18** within the ATP pocket of AURKA was conducted using the reported imidazo[4,5-*b*]pyridine-AURKA complex (PDB code: 4BYI) as the starting point (Figure 2, panel B)^21^. Similar to modeling studies with EGFR, one hydrogen bond to the hinge region and interactions with the adenine region of the ATP pocket was defined as the key pharmacophore for binding to AURKA. A comparison of the docked pose for compounds **1**–**18** and the bound pose for imidazo[4,5-*b*]pyridine within AURKA demonstrated that the 4-anilino moiety did not overlay on the pyrazole or pyrrolidine moieties of the crystallised imidazo[4,5-*b*]pyridine. The 4-anilino moiety was solvent exposed and did not bind within the hydrophobic region or the sugar pocket of the ATP site of AURKA.

Figure 2.Molecular modeling for compounds **1–18** within the ATP pocket of EGFR and AURKA Panel A: an overlay of the docked poses of compounds **1–7** and **9–15** on a reported pyrimido[4,5-b]azepine in EGFR (left panel), compound **2** in EGFR showing interactions with the backbone of Met793 in the hinge region, adenine region and hydrophobic region I (right panel). Panel B: an overlay of the docked poses of compounds **1–7** and **9–15** on a reported imidazo[4,5-b]pyridine in AURKA (left panel), compound 2 in AURKA showing interactions with the backbone of Ala213 in the hinge region and adenine region (right panel). Panel C: an overlay of the docked poses of compounds **2** and **8** in EGFR (left panel) and AURKA (right panel). An additional hydrogen bond is observed with the backbone of Gln791 of the hinge region of AURKA for compound **8**. Panel D: an overlay of the docked poses of compounds **16, 17** and **18** in EGFR (left panel) and AURKA (right panel).

**Figure:**
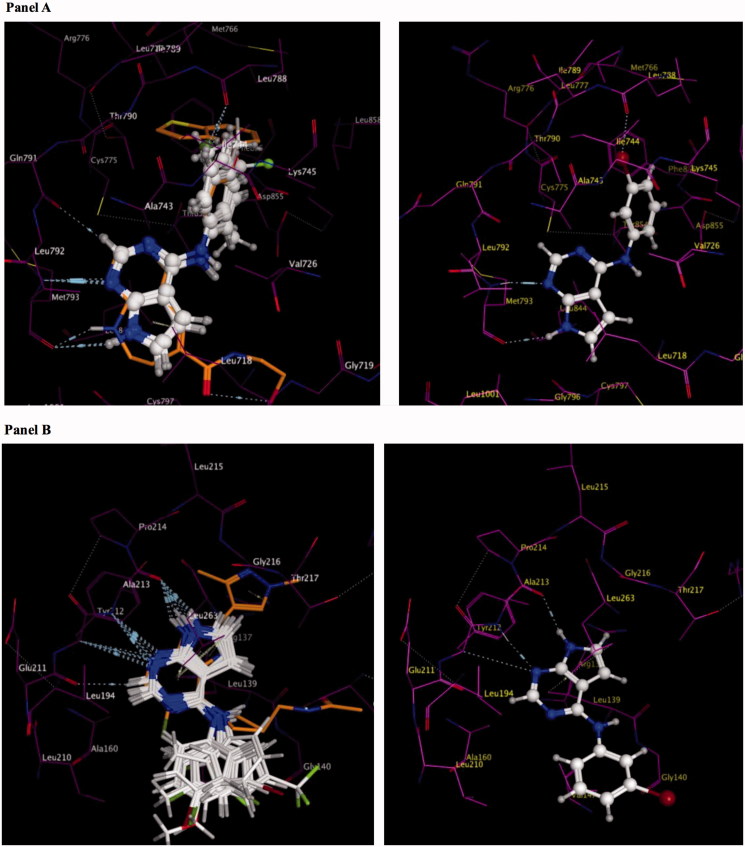

**Figure:**
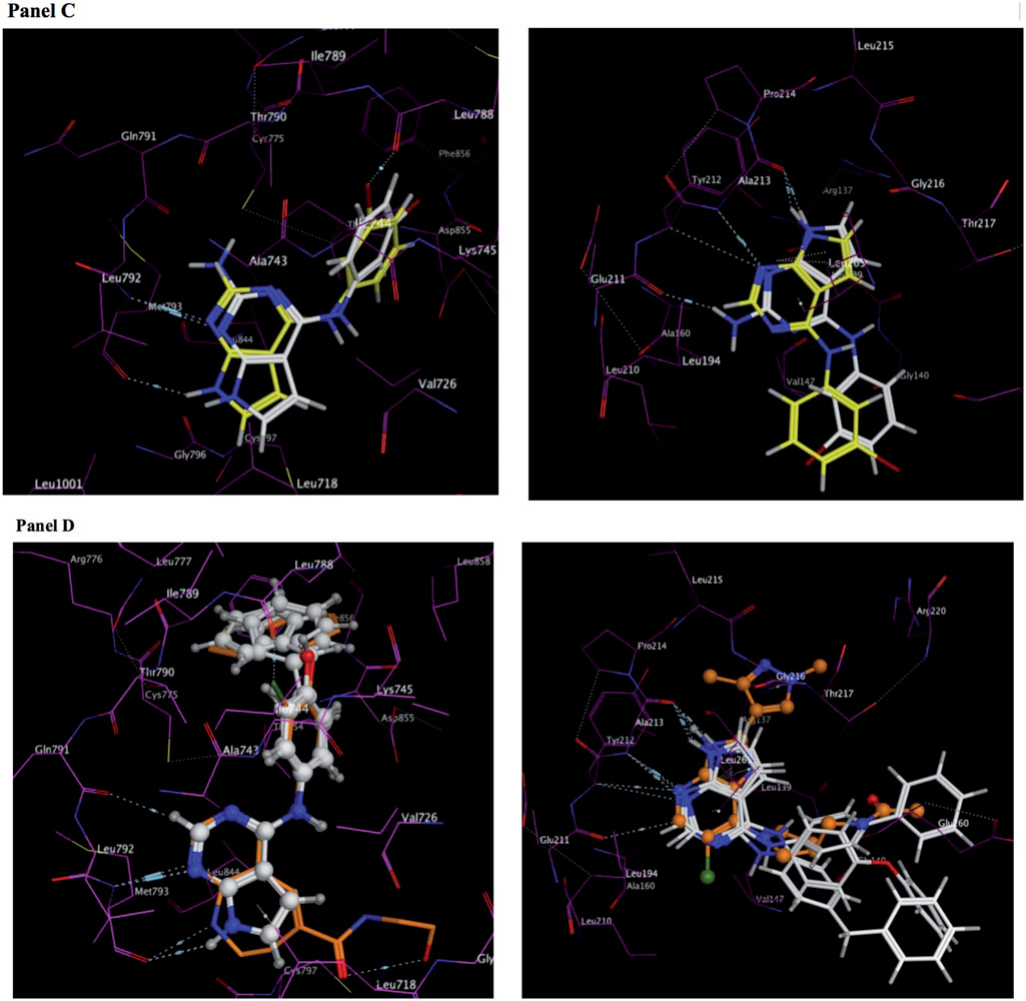

Compounds **5** and **7** that merged potent 3′-substitutions with 4′-substitutions on the 4-anilino moiety were found to be accommodated in both EGFR and AURKA with a similar mode of binding as seen for **2**. Compound **8** of this study was designed as a 2-amino substituted derivative of compound **2** to evaluate whether potency for kinase inhibition could be improved by incorporating an additional hydrogen bond to the hinge region. Molecular modeling for compound **8** revealed an additional hydrogen bond to the hinge region of AURKA but not for EGFR (Figure 2, panel C). Larger substitutions such as 4-phenoxy seen in **17**, 4-benzyl in **18** and 4-biphenyl substitutions in **16** were found to bind deeper in the hydrophobic region I for EGFR and overlaid on the benzothiophene ring of the reported EGFR inhibitor (Figure 2, panel D). The larger substitutions were found to extend towards the phosphate binding region in AURKA. Molecular modeling demonstrated better binding to EGFR versus AURKA for compounds **1**–**18**. It was of interest to determine if the results would translate to enzymatic inhibition.

**Scheme 1.**
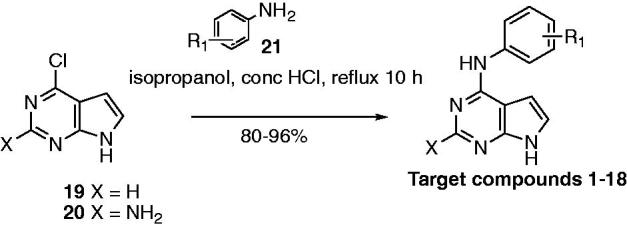
Synthesis of target compounds **1–18**.

For further evaluation against target enzymes and in SCCHN cells, compounds **1**–**18** were synthesised as described in Scheme 1. The synthetic procedure for some of the target compounds has been described as part of a patent; however, the experimental analysis has not been reported^15^^,^^16^. Compounds **1**–**18** were synthesised using a modification of the previously reported protocol^14^. Nucleophilic substitution of **19** or **20** with the appropriate aniline **21** in isopropanol and a few drops of conc HCl at reflux afforded the target compounds **1**–**18** in 80–96% yields. Compounds **1**–**18** were screened against AURKA and EGFR. Potent inhibitors were further evaluated in SCCHN cells.

## Methods

### Molecular modeling

Compounds **1**–**18** were docked within the ATP binding site of EGFR and AURKA using reported crystal structures for EGFR (PDB code: 3W33) and AURKA (PDB code: 4BYI) as the starting points for the molecular modeling studies and the computational software, Molecular Operating Environment (MOE 2016.08) suite^20–22^. A pharmacophore query was created for EGFR and AURKA using annotation points such as aromatic centres, H-bond donors and acceptors, and hydrophobic centres of the crystallised ligands. The crystallised ligands were imidazo[4,5-*b*]pyridine in AURKA and pyrimido[4,5-*b*]azepine in EGFR. Compounds **1**–**18** were created in MOE using the molecule builder function in MOE and were energy minimised using the MMF94X forcefield to a constant of 0.05 kcal/mol. Ligands were docked into the active site of the protein using the docking suite function in MOE. The docking was restricted to the active site pocket residues using the pharmacophore placement method. Refinement of the docked poses was carried out using the Forcefield refinement scheme and scored using Affinity dG. The docked poses were examined for fit and interactions within the binding pocket using LigX within MOE.

#### Synthesis

Analytical samples were dried *in vacuo* (0.2 mmHg) over P_2_O_5_. Nuclear magnetic resonance spectra for proton (^1^H NMR) were recorded on a Bruker (400 MHz) spectrometer. The chemical shift values are expressed in parts per million (ppm) relative to tetramethylsilane as an internal standard: s, singlet; d, doublet; t, triplet; q, quartet; m, multiplet; and br, broad singlet. Mass spectra were recorded on a VG-7070 double-focusing mass spectrometer or in a LKB-9000 instrument in the electron ionisation (EI) or electron spray (ESI) mode. Chemical names follow IUPAC nomenclature. Thin-layer chromatography (TLC) was performed on Whatman Sil G/UV254 silica gel plates with a fluorescent indicator, and the spots were visualised under 254 and 366 nm illumination. Proportions of solvents used for TLC are by volume. Column chromatography was performed on an Isolera Prime system with 254 nm detector (Biotage, Charlotte, NC, USA) utilizing 230–400 mesh silica gel snap cartridges. All solvents and chemicals were purchased from Aldrich, USA or VWR Scientific, USA and were used as received.

#### *N*^4^-phenyl-7*H*-pyrrolo[2,3-*d*]pyrimidine-4-amine (1)

A 100-ml round-bottom flask was added **19** (300 mg, 1.95 mmol), aniline, **21** (1.2 eq), *i*PrOH (20 ml) and six drops of conc HCl. The mixture was refluxed for 10 h. After being cooled, the reaction mixture was dried *in vacuo*. The residue was neutralised with NH_4_OH (1 ml) and extracted with CH_2_Cl_2_ (30 ml). The organic layer was dried over Na_2_SO_4_, filtered, and concentrated under reduced pressure to afford a yellow solid. The crude product was purified by flash chromatography on silica gel (gradient, CH_2_Cl_2_ to 10% of MeOH/CH_2_Cl_2_) to afford 400 mg (84%) of **1** as a white solid, TLC *R_f_* 0.53 (CH_2_Cl_2_/CH_3_OH, 10:1), ^1^H NMR (DMSO-d_6_) δ 6.76–6.77 (m, *J* = 4 Hz, 1 H), 6.98 (t, *J* = 12 Hz, 2 H), 7.20–7.21 (m, *J* = 4 Hz, 1 H), 7.31 (t, *J* = 12 Hz, 2 H), 7.86 (d, *J* = 12 Hz, 2 H), 8.25 (s, 1 H), 9.26 (s, 1 H), ^13^ C NMR (400 MHz DMSO-d_6_) δ 153.96, 151.37, 151.19, 140.97, 129.62, 122.56, 120.69, 104.20, 99.22); HRMS (ESI) (M + H)^+^: Calcd for C_12_H_11_N_4_*m/z* = 211.0978, found *m/z* = 211.0978.

#### *N*^4^-(3-bromophenyl)-7*H*-pyrrolo[2,3-*d*]pyrimidine-4-amine (2)

Compound **2** was synthesised as described for **1** with 3-bromoaniline and was obtained as a white solid (82%), TLC *R_f_* 0.58 (CH_2_Cl_2_/CH_3_OH, 10:1), ^1^H NMR (400 MHz DMSO-d_6_) δ 6.79–6.80 (m, *J* = 4 Hz, 1 H), 7.15 (d, *J* = 8 Hz, 1 H), 7.25–7.29 (m, 2 H), 7.86 (d, *J* = 8 Hz, 1 H), 8.31–8.32 (m, 2 H), 9.44 (s, 1 H), ^13^C NMR (400 MHz DMSO-d_6_) δ 153.53, 151.39, 151.04, 142.67, 130.87, 124.63, 123.11, 122.32, 121.85, 118.90, 104.37, 99.09; HRMS (ESI) (M + H)^+^: Calcd for C_12_H_10_N_4_Br *m/z* = 289.0089, found *m/z* = 289.0075.

#### *N*^4^-(3-fluoro-4-chlorophenyl)-7*H*-pyrrolo[2,3-*d*]pyrimidine-4-amine (3)

Compound **3** was synthesised as described for **1** with 3-fluoro-4-chloroaniline and was obtained as a white solid (87%), TLC *R_f_* 0.59 (CH_2_Cl_2_/CH_3_OH, 10:1), ^1^H NMR (400 MHz DMSO-d_6_) δ 6.76–6.77 (m, *J* = 4 Hz, 1 H), 7.25–7.26 (m, *J* = 4 Hz, 1 H), 7.36 (t, 1 H), 7.77–7.81 (m, 2 H), 8.27–8.31 (m, 2 H), 9.46 (s, 1 H), ^13^C NMR (400 MHz DMSO-d_6_) δ 154.15, 153.51, 151.33, 151.02, 138.26, 123.07, 121.44, 120.37, 119.31, 117.12, 104.17, 99.03; HRMS (ESI) (M + H)^+^: Calcd for C_12_H_9_N_4_FCl *m/z* = 263.0422, found *m/z* = 263.0483.

#### *N*^4^-(4-chlorophenyl)-7*H*-pyrrolo[2,3-*d*]pyrimidine-4-amine (4)

Compound **4** was synthesised as described for **1** with 4-chloroaniline and was obtained as a white solid (86%), TLC *R_f_*0.59 (CH_2_Cl_2_/CH_3_OH, 10:1), ^1^H NMR (400 MHz DMSO-d_6_) δ 6.79–6.80 (m, *J* = 4 Hz, 1 H), 7.25–7.26 (m, *J* = 4 Hz, 1 H), 7.37(d, *J* = 12 Hz, 2 H), 7.95 (d, *J* = 12 Hz, 2 H), 8.29 (s, 1 H), 9.44 (s, 1 H); ^13^C NMR (400 MHz DMSO-d_6_) δ 153.63, 151.30, 151.02, 139.89, 128.77, 125.81, 122.91, 121.91, 104.24, 99.18; HRMS (ESI) (M + H)^+^: Calcd for C_12_H_10_ClN_4_*m/z* = 245.0589, found *m/z* = 245.0588.

#### *N*^4^-(3,4-dichlorophenyl)-7*H*-pyrrolo[2,3-*d*]pyrimidine-4-amine (5)

Compound **5** was synthesised as described for **1** with 3,4-dichloroaniline and was obtained as a white solid (80%), TLC *R_f_* 0.59 (CH_2_Cl_2_/CH_3_OH, 10:1), ^1^H NMR (400 MHz DMSO-d_6_) δ 6.78 (d, *J* = 4 Hz, 1 H), 7.27 (d, *J* = 4 Hz, 1 H), 7.53 (d, 1 H), 7.85–7.88 (m, *J* = 12 Hz, 1 H), 8.34 (s, 1 H), 8.39 (d, 1 H), 9.54 (s, 1 H), ^13^C NMR (400 MHz DMSO-d_6_) δ 153.31, 151.45, 150.95, 141.23, 131.12, 130.78, 123.33, 121.07, 120.06, 104.47, 99.01; HRMS (ESI) (M + H)^+^: Calcd for C_12_H_9_N_4_Cl_2_*m/z* = 279.0199, found *m/z* = 279.0198.

#### *N*^4^-(4-methoxyphenyl)-7*H*-pyrrolo[2,3-*d*]pyrimidine-4-amine (6)

Compound **6** was synthesised as described for **1** with 4-methoxyaniline and was obtained as a white solid (90%), TLC *R_f_* 0.52 (CH_2_Cl_2_/CH_3_OH, 10:1), ^1^H NMR (400 MHz DMSO-d_6_) δ 3.73 (s, 3 H), 6.64 (d, *J* = 4 Hz, 1 H), 6.91 (d, *J* = 8 Hz, 2 H), 7.15–7.16 (m, *J* = 4 Hz, 1 H), 7.69 (d, *J* = 8 Hz, 2 H), 8.18 (s, 1 H), 9.12 (s, 1 H), ^13^C NMR (400 MHz DMSO-d_6_) δ 155.26, 154.30, 151.36, 151.18, 133.76, 122.89, 122.16, 114.15, 103.66, 99.23; HRMS (ESI) (M + H)^+^: Calcd for C_13_H_13_N_4_O *m/z* = 241.1084, found *m/z* = 241.1083.

#### *N*^4^-(3-bromo, 4-chlorophenyl)-7*H*-pyrrolo[2,3-*d*]pyrimidine-4-amine (7)

Compound **7** was synthesised as described for **1** with 3-bromo-4-chloroaniline and was obtained as a white solid (82%); TLC *R_f_* 0.59 (CH_2_Cl_2_/CH_3_OH, 10:1), ^1^H NMR (400 MHz DMSO-d_6_) δ 6.82 (d, *J* = 4 Hz, 1 H), 7.28 (d, *J* = 4 Hz, 1 H), 7.54 (d, *J* = 12 Hz, 1 H), 7.91–7.94 (m, *J* = 12 Hz, 1 H), 8.35 (s, 1 H, CH), 8.47 (d, 1 H), 9.52 (s, 1 H, NH); ^13^C NMR (400 MHz DMSO-d_6_) δ 153.16, 150.96, 150.39, 140.87, 130.62, 125.70, 124.62, 123.48, 121.48, 121.01, 104.41, 99.35; HRMS (ESI) (M + H)^+^: Calcd for C_12_H_9_N_4_BrCl *m/z* = 322.9694, found *m/z* = 322.9693.

#### *N*^4^-(3-bromophenyl)-7*H*-pyrrolo[2,3-*d*]pyrimidine-2,4-diamine (8)

Compound **8** was synthesised as described for **1** and 3-bromoaniline and was obtained as a white solid (81%), TLC *R_f_* 0.50 (CH_2_Cl_2_/CH_3_OH, 10:1), ^1^H NMR (400 MHz DMSO-d_6_) δ 5.78 (s, 2 H), 6.54–6.55 (m, *J* = 4 Hz, 1 H), 6.77–6.78 (m, *J* = 4 Hz, 1 H), 7.09 (d, *J* = 12 Hz, 1 H), 7.20–7.23 (m, *J* = 12 Hz, 1 H), 8.04 (d, *J* = 12 Hz, 1 H), 8.14 (s, 1 H), 9.04 (s, 1 H), 10.88 (s, 1 H); ^13^ C NMR (400 MHz DMSO-d_6_) δ 155.61, 153.88, 140.63, 130.95, 127.14, 124.75, 121.79, 121.41, 119.96, 102.10, 98.03; HRMS (ESI) (M + H)^+^: Calcd for C_12_H_10_BrN_5_*m/z* = 303.0120, found *m/z* = 303.0114.

#### *N*^4^-(4-methylphenyl)-7*H*-pyrrolo[2,3-*d*]pyrimidin-4-amine (9)

Compound **9** was synthesised as described for **1** with 4-methylaniline and was obtained as a white solid (94%); TLC *R_f_* 0.57 (CH_2_Cl_2_/CH_3_OH, 10:1); ^1^H NMR (400 MHz, DMSO-d_6_) δ 6.86 (d, *J* = 4 Hz, 1 H), 7.27 (d, *J* = 8 Hz, 2 H), 7.37 (d, *J* = 4 Hz, 1 H), 7.46 (d, *J* = 8 Hz, 2 H), 8.30 (s, 1 H), 11.33 (s, 1 H); ^13^ C NMR (400 MHz DMSO-d_6_) δ150.79, 147.29, 144.13, 136.51, 133.91, 130.41, 125.05, 124.85, 102.70, 21.09; HRMS (ESI) (M + H)^+^: Calcd for C_13_H_13_N_4_*m/z* = 225.1062, found *m/z* = 225.1084.

#### *N*^4^-(4-bromophenyl)-7*H*-pyrrolo[2,3-*d*]pyrimidin-4-amine (10)

Compound **10** was synthesised as described for **1** with 4-bromoaniline and was obtained as a white solid (91%); TLC *R_f_* 0.60 (CH_2_Cl_2_/CH_3_OH, 10:1); ^1^H NMR (400 MHz, DMSO-d_6_) δ 7.04 (d, *J* = 4 Hz, 1 H), 7.41 (d, *J* = 4 Hz, 1 H), 7.62–7.65 (m, 4 H), 8.37 (s, 1 H), 11.40 (s, 1 H); ^13^ C NMR (400 MHz DMSO-d_6_) δ 151.41, 146.33, 144.64, 136.55, 132.62, 126.42, 125.04, 118.73, 103.46, 102.50; HRMS (ESI) (M + H)^+^: Calcd for C_12_H_10_BrN_4_*m/z* = 289.0083, found *m/z* = 289.0082.

#### *N*^4^-(3-chlorophenyl)-7*H*-pyrrolo[2,3-*d*]pyrimidin-4-amine (11)

Compound **11** was synthesised as described for **1** with 3-chloroaniline and was obtained as a white solid (95%); TLC *R_f_* 0.68 (CH_2_Cl_2_/CH_3_OH, 10:1); ^1^H NMR (400 MHz, DMSO-d_6_) δ 6.80 (d, *J* = 4 Hz, 1 H), 7.01 (d, *J* = 8 Hz, 1 H), 7.02 (d, *J* = 4 Hz, 1 H), 7.30 (t, 1 H), 7.79 (d, *J* = 8 Hz, 1 H), 8.21 (s, 1 H), 8.34 (s, 1 H), 9.44 (s, 1 H); ^13^C NMR (400 MHz DMSO-d_6_) δ 153.58, 151.43, 151.04, 142.53, 133.32, 130.51, 123.07, 121.74, 119.56, 118.54, 104.40, 99.11; HRMS (ESI) (M + H)^+^: Calcd for C_12_H_10_N_4_Cl *m/z* = 245.0516, found *m/z* = 245.0582.

#### *N*^4^-(3-methylaniline)-7*H*-pyrrolo[2,3-*d*]pyrimidin-4-amine (12)

Compound **12** was synthesised as described for **1** with 3-methylaniline and was obtained as a white solid (94%); TLC *R_f_* 0.54 (CH_2_Cl_2_/CH_3_OH, 10:1); ^1^H NMR (400 MHz, DMSO-d_6_) δ 2.30 (s, 3 H), 6.80–6.82 (m, 2 H), 7.19–7.21 (m, 2 H), 7.68–7.22 (m, 2 H), 8.27 (s, 1 H), 9.19 (s, 1 H); ^13^C NMR (400 MHz DMSO-d_6_) δ154.05, 151.30, 151.25, 140.79, 137.97, 128.75, 123.20, 122.48, 121.18, 117.95, 104.11, 99.25, 21.74; HRMS (ESI) (M + H)^+^: Calcd for C_13_H_13_N_4_*m/z* = 225.1062, found *m/z* = 225.1134.

#### *N*^4^-(3-trifluoromethylphenyl)-7*H*-pyrrolo[2,3-*d*]pyrimidin-4-amine (13)

Compound **13** was synthesised as described for **1** with 3-trifluoromethylaniline and was obtained as a white solid (92%); TLC *R_f_* 0.70 (CH_2_Cl_2_/CH_3_OH, 10:1); ^1^H NMR (400 MHz, DMSO-d_6_) δ 6.81 (d, *J* = 4 Hz, 1 H), 7.27–7.31 (m, 2 H), 7.54 (t, 1 H), 8.23 (d, *J* = 8 Hz, 1 H), 8.34 (s, 1 H), 8.38 (s, 1 H), 9.59 (s, 1 H); ^13^C NMR (400 MHz DMSO-d_6_) δ153.58, 151.45, 151.01, 141.84, 130.04, 129.90, 129.58, 123.60, 123.19, 118.27, 118.23, 116.15, 116.11, 104.43, 99.07; HRMS (ESI) (M + H)^+^: Calcd for C_13_H_10_N_4_F_3_*m/z* = 279.0779, found *m/z* = 279.0851.

#### *N*^4^-(2-chlorophenyl)-7*H*-pyrrolo[2,3-*d*]pyrimidin-4-amine (14)

Compound **14** was synthesised as described for **1** with 2-chloroaniline and was obtained as a white solid (81%); TLC *R_f_* 0.61 (CH_2_Cl_2_/CH_3_OH, 10:1); ^1^H NMR (400 MHz, DMSO-d_6_) δ 6.49 (d, *J* = 4 Hz, 1 H), 7.17 (d, *J* = 4 Hz, 1 H), 7.23 (t, 1 H), 7.33–7.37 (t, 1 H), 7.52 (d, *J* = 8 Hz, 1 H), 7.68 (d, *J* = 8 Hz, 1 H), 8.12 (s, 1 H), 9.02 (s, 1 H); ^13^C NMR (400 MHz DMSO-d_6_) δ 154.80, 151.61, 151.32, 136.89, 130.02, 129.83, 128.97, 127.82, 126.90, 122.63, 103.59, 99.17; HRMS (ESI) (M + H)^+^: Calcd for C_12_H_10_ClN_4_*m/z* = 245.0516, found *m/z* = 245.0588.

#### *N*^4^-(2-methylphenyl)-7*H*-pyrrolo[2,3-*d*]pyrimidin-4-amine (15)

Compound **15** was synthesised as described for **1** with 2-methylaniline and was obtained as a white solid (83%); TLC *R_f_* 0.57 (CH_2_Cl_2_/CH_3_OH, 10:1); ^1^H NMR (400 MHz, DMSO-d_6_) δ 2.19 (s, 3 H), 6.27 (d, *J* = 4 Hz, 1 H), 7.10 (d, *J* = 4 Hz, 1 H), 7.13–7.26 (m, 3 H), 7.36 (d, *J* = 8 Hz, 1 H), 8.09 (s, 1 H), 8.87 (s, 1 H); ^13^C NMR (400 MHz DMSO-d_6_) δ 155.51, 151.60, 151.53, 138.26, 134.71, 130.78, 127.62, 126.50, 125.99, 122.00, 103.14, 99.34, 18.50; HRMS (ESI) (M + H)^+^: Calcd for C_13_H_13_N_4_*m/z* = 225.1062, found *m/z* = 225.1134.

#### *N*^4^-(4-biphenyl)-7*H*-pyrrolo[2,3-*d*]pyrimidin-4-amine (16)

Compound **16** was synthesised as described for **1** with 4-aminobiphenyl and was obtained as a white solid (89%); TLC *R_f_* 0.65 (CH_2_Cl_2_/CH_3_OH, 10:1); ^1^H NMR (400 MHz, DMSO-d_6_) δ 6.84 (d, *J* = 4 Hz, 1 H), 7.25–7.31 (m, 2 H), 7.42 (d, *J* = 8 Hz, 2 H), 7.64–7.66 (m, 4 H), 8.01 (d, *J* = 8 Hz, 2 H), 8.32 (s, 1 H), 9.47 (s, 1 H); ^13^C NMR (400 MHz DMSO-d_6_) δ153.79, 151.27, 151.03, 140.39, 140.35, 134.10, 129.32, 127.23, 127.14, 126.58, 122.78, 120.99, 104.26, 99.34; HRMS (ESI) (M + H)^+^: Calcd for C_18_H_15_N_4_*m/z* = 287.1218, found *m/z* = 287.5214.

#### *N*^4^-(4-phenoxyphenyl)-7*H*-pyrrolo[2,3-*d*]pyrimidin-4-amine (17)

Compound **17** was synthesised as described for **1** with 4-phenoxyaniline and was obtained as a white solid (96%); TLC *R_f_* 0.66 (CH_2_Cl_2_/CH_3_OH, 10:1); ^1^H NMR (400 MHz, DMSO-d_6_) δ 6.75 (d, *J* = 4 Hz, 1 H), 7.02–7.09 (m, 5 H), 7.21 (d, *J* = 4 Hz, 1 H), 7.35 (t, 2 H), 7.88 (d, *J* = 8 Hz, 2 H), 8.25 (s, 1 H), 9.31 (s, 1 H); ^13^C NMR (400 MHz DMSO-d_6_) δ158.13, 153.98, 151.27, 151.24, 136.85, 130.36, 123.20, 122.51,122.42, 119.89, 118.09, 103.96, 99.20; HRMS (ESI) (M + H)^+^: Calcd for C_18_H_15_ON_4_*m/z* = 303.1168, found *m/z* = 303.1240.

#### *N*^4^-(4-benzylphenyl)-7*H*-pyrrolo[2,3-*d*]pyrimidin-4-amine (18)

Compound **18** was synthesised as described for **1** with 4-benzylaniline and was obtained as a white solid (93%); TLC *R_f_* 0.63 (CH_2_Cl_2_/CH_3_OH, 10:1); ^1^H NMR (400 MHz, DMSO-d_6_) δ 3.97 (s, 2 H), 6.92 (d, *J* = 4 Hz, 1 H), 7.18–7.29 (m, 5H), 7.33 (d, *J* = 8 Hz, 2H), 7.38 (d, *J* = 4 Hz, 1 H), 7.50 (d, *J* = 8 Hz, 2 H), 8.29 (s, 1 H), 11.34 (s, 1 H); ^13^C NMR (400 MHz DMSO-d_6_) δ150.74, 147.08, 144.21, 141.46, 140.19, 134.46, 130.15, 129.19, 128.93, 126.51, 124.96, 124.92, 102.65, 41.06. HRMS (ESI) (M + H)^+^: Calcd for C_19_H_17_N_4_*m/z* = 301.1375, found *m/z* = 301.3691.

#### Kinase assay

The assay was performed externally at BPS Biosciences using the ADP-Glo Kinase assay (23). Kinase activity was measured by quantifying the amount of ADP produced from the kinase reaction. The luminescent signal from the assay was correlated with the amount of ADP present and is directly correlated with the amount of kinase activity. Compounds **1**–**18** and staurosporine were diluted in 10% of DMSO and 2.5 µl of the dilution was added to a 25 µl of reaction so that the final concentration of DMSO is 1% in all of reactions. The 25 µl of reaction mixture contained 40 mM Tris, pH 7.4, 10 mM MgCl_2_, 0.1 mg/ml BSA, 1 mM DTT, 10 µM ATP, kinase substrate peptide, 10 μM ATP and the target kinase enzyme. EGFR reactions were conducted at 30 °C for 45 min using 0.2 mg/ml Poly(Glu, Tyr) as the EGFR substrate peptide and 5 ng of EGFR kinase. Aurora A reactions were performed for 50 min at 30 °C using 0.2 mg/ml Kemptide and 220 ng of aurora A kinase. After the kinase reaction at 30 °C, 25 µl of ADP-Glo reagent was added and incubated for 45 min at room temperature followed by another 40 min incubation with 50 µl of kinase detection mixture. All luminescence signals were measured using a BioTek *Synergy 2* microplate reader. Kinase activity assays were performed in triplicate at each concentration. The luminescence data were analysed using the computer software, Graphpad Prism 6.0 (GraphPad Software Inc., La Jolla, CA, USA).

#### Binding affinities for EGFR, AURKA and AURKB

The assay was performed externally at DiscoverX Corporation using a competition binding assay that quantitatively measures the ability of a compound to compete with an immobilised, active-site directed ligand^24^. The assay is performed by combining three components: DNA-tagged kinase, immobilised ligand and a test compound. The ability of the test compound to compete with the immobilised ligand was measured via quantitative PCR of the DNA tag. An 11-point 3-fold serial dilution of each test compound was prepared in 100% of DMSO at 100× final test concentration and subsequently diluted to 1× in the assay (final DMSO concentration = 1%). Compound K_d_ was determined using a compound top concentration = 30,000 nM. If the initial K_d_ determined was <0.5 nM (the lowest concentration tested), the measurement was repeated with a serial dilution starting at a lower top concentration. Binding constants (K_d_) were calculated with a standard dose-response curve using the Hill equation.

#### Proliferation and cell killing assays in SCCHN cells

FADU, BHY, SAS and CAL cell lines were obtained from ATCC-LGC and were cultured in DMEM (Invitrogen, Germany) supplemented with 10% of heat activated bovine serum (FBS, PAA, Germany), 1% of glutamine, 1% of penicillin-streptomycin (Invitrogen, Germany). To measure proliferation, SCCHN cells were split, reseeded (5 × 10^5^ in 25 cm^2^ flasks) and counted at the indicated time points. Cells were then replated at the initial density. The fold increase in cell number was calculated, all given results are based on triplicate experiments. To assess cell death 5 × 10^5^ cells were stained with propidium iodide (PI, Sigma, Germany). Following incubation, cells were washed, resuspended in PBS, and analysed by flow cytometry. The fraction of PI-positive cells is reported as dead cell fraction.

#### Western blot analysis of EGFR and aurora kinase downstream target proteins

Protein extracts (50 μg per lane) were electrophoretically separated on SDS-PAGE gels, transferred to membranes (Protran, Schleicher & Schuell, Dassel, Germany) and blotted with specific antibodies (actin, aurora A, aurora B: all from Sigma, Munich, Germany; S10-HH3: Millipore, Schwalbach, Germany; EGFR: Santa Cruz, Heidelberg, Germany; pEGFR: Invitrogen, Darmstadt, Germany; pAKT, pERK: both from New England Biolabs, Frankfurt, Germany).

#### Cell cycle analysis

For analysis of cell cycle distribution, cells were fixed with 70% of ethanol and stained with PI. Flow cytometric analysis of DNA content was performed using PI in the FL2 chanel in linear mode. Cells with less than diploid DNA content were considered dead (sub-G1).

## Results

Compounds **1**–**18** were screened against AURKA and EGFR using the ADP-Glo luminescence assay (BPS Bioscience Inc., San Diego, CA, USA)^23^ at final concentrations ranging from 3 nM to 100 μM and were evaluated in triplicate. A known kinase inhibitor, staurosporine was included for comparison in this study. Inhibitory data against AURKA and EGFR are reported in Table 1.

**Table 1.** IC_50_ values for target compounds against AURKA and EGFR.

Targetcompound	R1	X	AURKA IC_50_ ± SD	EGFR IC_50_ ± SD
**1**	H	H	5.58 ± 0.66 μM	254.13 ± 25.97 nM
**2**	3-Br	H	1.99 ± 0.05 μM	3.76 ± 0.12 nM
**3**	3-F, 4-Cl	H	3.29 ± 0.15 μM	5.98 ± 0.61 nM
**4**	4-Cl	H	5.15 ± 0.37 μM	84.92 ± 15.92 nM
**5**	3,4-diCl	H	3.91 ± 0.11 μM	6.70 ± 0.25 nM
**6**	4-OCH_3_	H	6.68 ± 0.54 μM	2936.67 ± 94.40 nM
**7**	3-Br, 4-Cl	H	3.23 ± 0.31 μM	3.63 ± 0.43 nM
**8**	3-Br	NH_2_	4.54 ± 0.93 μM	383.7 ± 65.50 nM
**9**	4-CH_3_	H	5.45 ± 0.36 μM	667.13 ± 141.53 nM
**10**	4-Br	H	3.73 ± 0.12 μM	125.33 ± 16.44 nM
**11**	3-Cl	H	3.13 ± 0.75 μM	6.63 ± 0.98 nM
**12**	3-CH_3_	H	3.43 ± 0.12 μM	20.01 ± 3.60 nM
**13**	3-CF_3_	H	5.78 ± 0.43 μM	43.57 ± 7.77
**14**	2-Cl	H	5.66 ± 0.43 μM	470.67 ± 114.47 nM
**15**	2-CH_3_	H	8.56 ± 1.74 μM	1230 ± 201.12 nM
**16**	4-BiPh	H	74.36 ± 25.25 μM	>100 μM
**17**	4-OPh	H	13.27 ± 3.57 μM	110.97 ± 15.14 nM
**18**	4-CH_2_Ph	H	4.95 ± 0.69 μM	63.29 ± 4.58 nM
**Staurosporine**			0.46 ± 0.03 μM	430.57 ± 25.44 nM

**EGFR**: Compounds **2**, **3**, **5**, **7** and **11** demonstrated single-digit nanomolar EGFR inhibition. Compound **2** and **7** that included a 3′-bromo and a 3′-bromo, 4′-chloro substitution on the 4-anilino moiety respectively provided the most potent EGFR inhibition followed by compounds **3**, **5** and **11**. Compounds **2, 7** and **11** were over 140-fold more potent compared to the standard compound, staurosporine against EGFR. The 3′-monosubstituted and 3′,4′-disubstituted compounds demonstrated greater EGFR inhibition compared to the 4′-monosubstituted and 2′-monosubstituted compounds, respectively.

Compounds **17** and **18** with large 4-substitutions demonstrated EGFR inhibition that was comparable to other 4-substituted compounds of this series and were more potent than the unsubstituted compound **1**. An improvement in EGFR inhibitory potencies for **17** and **18** that was anticipated from molecular modeling results was not observed. Compound **16** incorporating the 4-biphenyl substitution demonstrated poor EGFR inhibition. Compounds with electron withdrawing groups on the 4-anilino moiety demonstrated greater EGFR inhibition compared to compounds incorporating electron donating groups. Compound **6** that included an electron-donating 4′-methoxy substitution demonstrated micromolar EGFR inhibition and was seven-fold less potent compared to staurosporine. Compound **17** incorporating a 4′-phenoxy substitution was also less potent than **18** incorporating the 4′-benzyl substitution indicating that incorporation of an oxygen atom at C-4 on the aniline moiety was not well tolerated. The incorporation of a 2-amino moiety in **8** did not improve EGFR inhibition. Compound **8** was found to be 100-fold less potent compared to compound **7**.

**AURKA**: Compounds **1**–**18** demonstrated single-digit micromolar inhibition for AURKA and were less potent compared to the standard compound, staurosporine. A similar trend was observed as seen for EGFR where compounds that included a 3′ or 3′,4′-disubstituted anilino moiety were slightly better than an unsubstituted, 4′-substituted or 2′-substituted anilino moiety, although the difference in AURKA inhibition was not as significant as was observed for EGFR. Compounds incorporating a 3′-substituted or 3′,4′-disubstituted anilino moiety were slightly better for AURKA compared to compound **1** incorporating an unsubstituted anilino moiety. Larger substitutions did not show better AURKA inhibition although compound **18** was approximately two-fold better than **17**. Compound **16** demonstrated poor AURKA inhibition. The 2-amino substituted compound **8** was approximately two-fold less potent compared to the parent compound **2** that lacked the 2-amino substitution.

Compound **2** was the most potent EGFR and AURKA inhibitor of the series. Due to the similarity in the structures of aurora kinases A and B, and a role for aurora kinase B in cancer progression and development of resistance, compound **2** was further evaluated for binding affinities for EGFR, AURKA and AURKB using a competition binding assay (DiscoverX Corporation, Fremont, CA, USA)^5^^,^^24^. Both AURKA and AURKB are implicated in tumour resistance pathways for selective EGFR inhibitors in cancer^25^. Binding constants (K_d_) for compound **2** were found to be 67 nM, 3.4 μM and 4.8 μM, respectively, for EGFR, AURKA and AURKB. Compound **2** demonstrated improved affinities for EGFR compared to AURKA. These results are consistent with the results of EGFR and AURKA inhibition from the luminescence assay.

**Figure 3.**
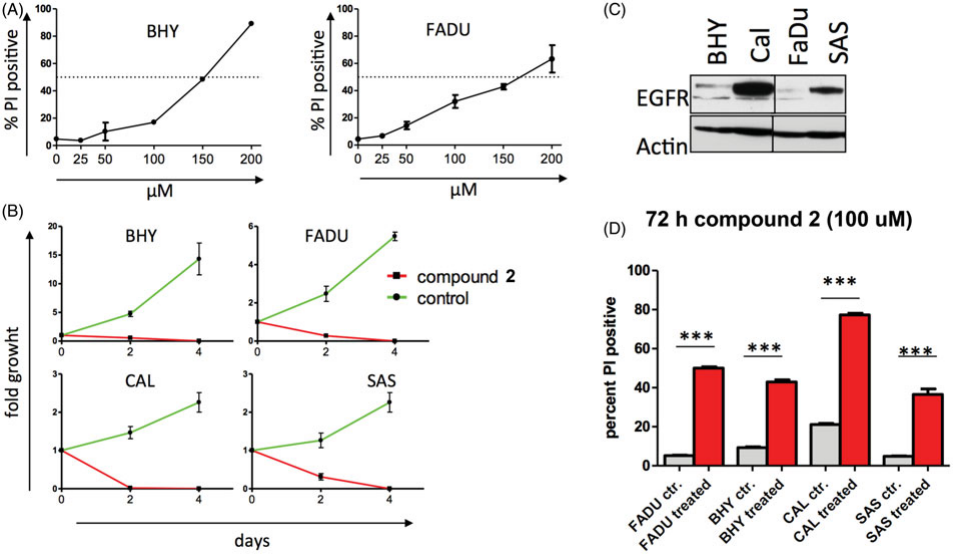
Compound **2** demonstrates antiproliferative effects in SCCHN cells (A) IC_50_ of BHY (EGFR low) and FADU (EGFR int) cell lines incubated with the indicated concentration of compound **2**. Cells were incubated with compound **2** for 48 h and then subjected to PI staining and FACS analysis. PI-positive cell fraction as measure of cell death is reported. (B) Four SCCHN cell lines were incubated with 100 μM of compound **2** and counted on the indicated days. Experiments in triplicates, fold growth shown. (C) EGFR expression of SCCHN cell lines. EGFR high and low as detected by western blot analysis (D) The SCCHN cell lines were incubated with 100 μM of compound **2** for 72 h. Percent PI positive cells are given. Experiments in triplicates.

Compound **2** was further evaluated for antiproliferative effects in SCCHN and to evaluate inhibitory effects of compound **2** on cell survival. Despite low EGFR expression, FADU cells are sensitive to cetuximab therapy and BHY cells are resistant to cetuximab treatment. On adding to cell culture media, compound **2** demonstrated modest cellular killing of BHY and FADU with IC_50_ concentrations of 150 µM (Figure 3, panel A). Interestingly, compound **2** completely inhibited cell growth at 100 µM of both BHY and FADU cells and two SCCHN cell lines (CAL and SAS) with marked overexpression of EGFR (Figure 3 (B,C)). Compound **2** demonstrated efficient cell killing at 100 µM concentrations in all four tested cell lines (Figure 3(D)).

**Figure 4.**
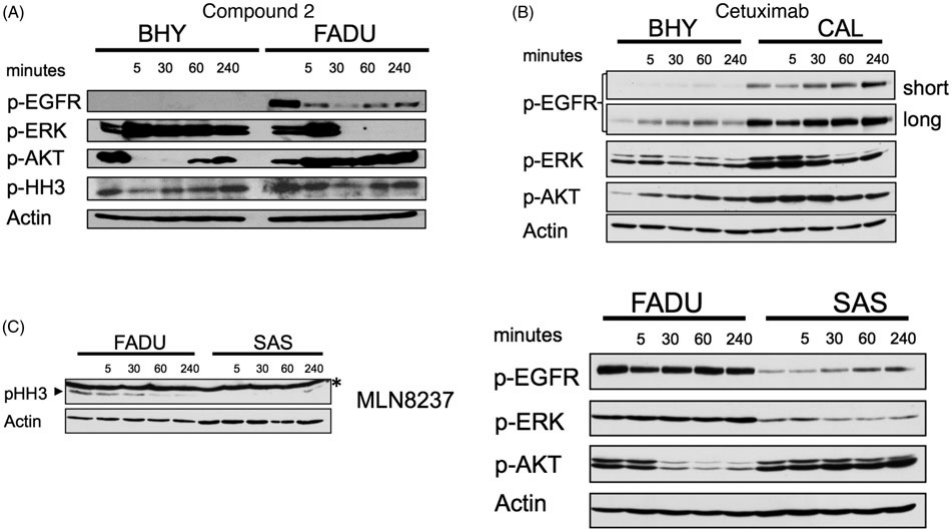
Effects of compound **2** on EGFR and aurora kinases in growing SCCHN cell lines (A) Western blot analysis of EGFR (pEGFR, pERK, p-AKT) and aurora A (p-HH3) downstream target proteins following incubation with 100 μM compound **2** for the indicated time points in BHY and FADU SCCHN cell lines. (B) BHY and CAL cell lines treated for the indicated time points with 200 nM cetuximab. Western blot analysis of EGFR downstream targets. (long exposure and short exposure). (C) FADU and SAS SCCHN cell lines incubated with 10 nM MLN8237 and 5 nM R736 showed inhibition of histone H3 phosphorylation. Western blot analysis of p-HH3 at the indicated time points. Asterisk (*) represents unspecific band.

**Figure 5.**
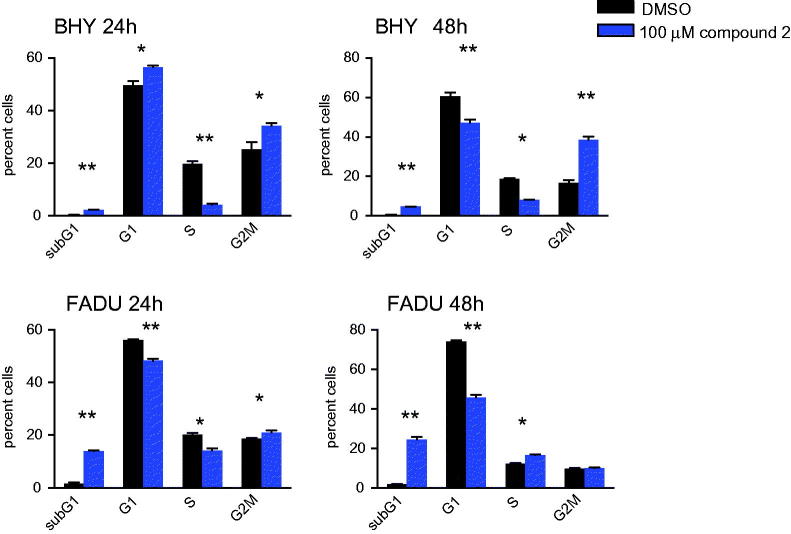
Cell cycle analysis of FADU and BHY cell lines incubated for 24 and 48 h with 100 μM of compound **2**. Cell cycle distribution according to PI uptake, shown is the cell fraction subG1 for cell death and in G1, S and G2M phase of the cell cycle. *n* = 3, * *p* < .01, ** *p* < .001.

To evaluate whether compound **2** was an inhibitor of EGFR and aurora kinases in growing cells, intracellular target inhibition was analysed (Figure 4). EGFR autophosphorylates and induces phosphorylation of downstream targets such as ERK and AKT. Initial hyperphosphorylation and later dephosphorylation of ERK and AKT have been reported for EGFR inhibition. All EGFR-mediated phosphorylation steps were modified by the addition of compound **2** to BHY cells and results were consistent with cetuximab treatment for the same time points (Figure 4(B))^10^. Downstream effects of EGFR blockade were found to be highly context and cell type specific (Figure 4(B)). Compound **2** was found to interfere with intracellular EGFR in SCCHN cell lines comparable to cetuximab. Phosphorylated histone H3 (p-HH3) served as a downstream target to assess intracellular effects mediated via aurora kinase inhibition, and was monitored as a direct target of AURKB and an indirect target of AURKA. Standard compounds MLN8237 and R736 were included for comparison. Modest effects on histone p-HH3 were observed for compound **2** as shown in Figure 4(A). These results are consistent with the modest inhibition and binding affinities observed for aurora kinases in the enzymatic assays. Interestingly during cell cycle analysis, compound **2** led to cell cycle arrest in G2M (more pronounced in BHY) followed by cell death (more pronounced in FADU) (Figure 5).

## Discussion

Compounds **1**–**18** demonstrated nanomolar EGFR inhibition and most compounds were more potent against EGFR compared to the standard compound, staurosporine. Compounds **1**–**18** demonstrated micromolar AURKA inhibition and were less potent against AURKA compared to staurosporine. These results were consistent with results from molecular modeling studies that indicated a 4-substituted pyrrolo[2,3-*d*]pyrimidine was accommodated in EGFR and AURKA, however improved binding interactions were observed for EGFR compared to AURKA. Significant differences in inhibitory potencies were observed for EGFR based on slight variations in the substitution pattern on the 4-anilino moiety. A preference for 3′-mono substituted or 3′,4′-disubstituted anilino moieties was observed compared to 4′- and 2′-substituted anilino moieties. A preference for electron withdrawing substitutions at the 4-anilino moiety was also observed. The 2-amino substitution incorporated in compound **8** did not improve EGFR and AURKA inhibition. A similar trend was observed for AURKA, although differences in activity were not as significant with variation in the substitution pattern of the 4-anilino moiety. Compound **6** demonstrated single-digit micromolar potencies against both EGFR and AURKA and was only 2-fold better for EGFR over AURKA. Compound **2** demonstrated the most potent EGFR and AURKA inhibition of this series in enzymatic assays and demonstrated antiproliferative effects against four different SCCHN cell lines at 100 micromolar concentrations. It was particularly noteworthy that inhibitory effects were seen for **2** in SCCHN cells irrespective of the EGFR status suggesting that inhibition of low levels of EGFR were significant if combined with aurora kinase inhibition. Compound **2** inhibited EGFR and aurora kinase mediated phosphorylation events in SCCHN cells. Modest cellular potencies for compound **2** could perhaps be explained by the high levels of ATP that were typically in the millimolar range for cells compared to micromolar concentrations used in the enzymatic assay. Compound **2** was a minimally functionalised molecule that did not explore many of the regions within the ATP binding site. The modest cellular inhibition for compound **2** underscores a need for more functionalised compounds that could bind to additional regions within the ATP binding site of both aurora kinases and EGFR, that could demonstrate potent cellular inhibition even at high levels of cellular ATP. Compound **2** and others identified through this study provide valuable leads for further functionalisation and development as inhibitors of EGFR and/or aurora kinase.

## Supplementary Material

IENZ_1376666_Supplementary_Material.pdf
