# Supplementary material for: Design, synthesis and biological activity of N4-phenylsubstituted-7H-pyrrolo[2,3-d]pyrimidin-4-amines as dual inhibitors of aurora kinase A and epidermal growth factor receptor kinase
Source: J Enzyme Inhib Med Chem. 2017 Nov 8;33(1):74–84. doi: 10.1080/14756366.2017.1376666 (PMC6009956; doi:10.1080/14756366.2017.1376666)
Supplement: IENZ_1376666_Supplementary_Material.pdf [file IENZ_A_1376666_SM9173.pdf]

**SUPPORTING INFORMATION**

# Design, synthesis and biological activity of *N*[4]-phenylsubstituted-7*H*-pyrrolo[2,3-*d*]pyrimidin-4-amines as dual inhibitors of aurora kinase A and epidermal growth factor receptor kinase

Sonali Kurup,[1]* Bradley McAllister,[1] Pavlina Liskova,[1] Trusha Mistry,[1] Anthony Fanizza,[2] Dan Stanford,[2] Jolanta Slawska,[3] Ulrich Keller,[3,4] Alexander Hoellein[3]

## General procedures for determination of $IC_{50}$ values for compounds 1-8

Kinase activity was measured by quantifying the amount of ADP produced from the kinase reaction. The luminescent signal from the assay was correlated with the amount of ADP present and is directly correlated with the amount of kinase activity. Compounds **1-18** and staurosporine were diluted in 10% DMSO and 2.5µl of the dilution was added to a 25µl reaction so that the final concentration of DMSO is 1% in all of reactions. The 25 µl reaction mixture contained 40 mM Tris, pH 7.4, 10 mM $MgCl_2$, 0.1 mg/ml BSA, 1 mM DTT, 10 µM ATP, Kinase substrate peptide, 10 µM ATP and the target kinase enzyme. EGFR reactions were conducted at 30 °C for 45 minutes using 0.2 mg/ml Poly (Glu, Tyr) as the EGFR substrate peptide and 5 ng of EGFR kinase. Aurora A reactions were done for 50 min at 30 °C using 0.2 mg/ml Kemptide and 220 ng of Aurora A kinase. After the kinase reaction at 30 °C, 25 µl of ADP-Glo reagent was added and incubated for 45 min at room temperature followed by another 40 min incubation with 50 µl of Kinase detection mixture. All luminescence signals were measured using a BioTek *Synergy 2* microplate reader. Kinase activity assays were performed in triplicate at each

concentration. The luminescence data were analyzed using the computer software, Graphpad Prism. The difference between luminescence intensities in the absence of Kinase ($Lu_t$) and in the presence of Kinase ($Lu_c$) was defined as 100 % activity ($Lu_t - Lu_c$). Using luminescence signal (Lu) in the presence of the compound, % activity was calculated as:

% activity = $\{(Lu_t - Lu)/(Lu_t - Lu_c)\}\times 100\%$, where Lu= the luminescence intensity in the presence of the compound. The values of % activity versus a series of compound concentrations were then plotted using non-linear regression analysis of Sigmoidal dose-response curve generated with the equation $Y=B+(T-B)/1+10^{((LogEC50-X)\times Hill\ Slope)}$, where Y=percent activity, B=minimum percent activity, T=maximum percent activity, X= logarithm of compound and Hill Slope=slope factor or Hill coefficient. The $IC_{50}$ value was determined by the concentration causing a half-maximal percent activity.

## 3.1. Results of the Effect of the Compounds on Kinases

### EGFR

**Table S1: Data for the Effect of SK-34 (compound 1) on EGFR Activity**

| SK-34 (Log [nM]) | EGFR Activity Luminescence | | | % Activity | | |
|---|---|---|---|---|---|---|
| | Set1 | Set2 | Set3 | Set1 | Set2 | Set3 |
| No Compound | 7698 | 7680 | 8361 | 97 | 97 | 106 |
| 0 | 8176 | 7755 | 8129 | 103 | 98 | 103 |
| 1.0 | 7979 | 8710 | 8392 | 101 | 110 | 106 |
| 1.5 | 7329 | 7208 | 7428 | 92 | 91 | 94 |
| 2.0 | 5247 | 5646 | 6009 | 66 | 71 | 76 |
| 2.5 | 3191 | 3411 | 3245 | 39 | 42 | 40 |
| 3.0 | 1985 | 1862 | 1823 | 24 | 22 | 22 |
| 3.5 | 811 | 792 | 802 | 9 | 9 | 9 |
| 4.0 | 446 | 549 | 482 | 4 | 5 | 5 |
| 4.5 | 257 | 265 | 275 | 2 | 2 | 2 |
| 5.0 | 201 | 208 | 222 | 1 | 1 | 1 |
| Background | 129 | 121 | 131 | | | |

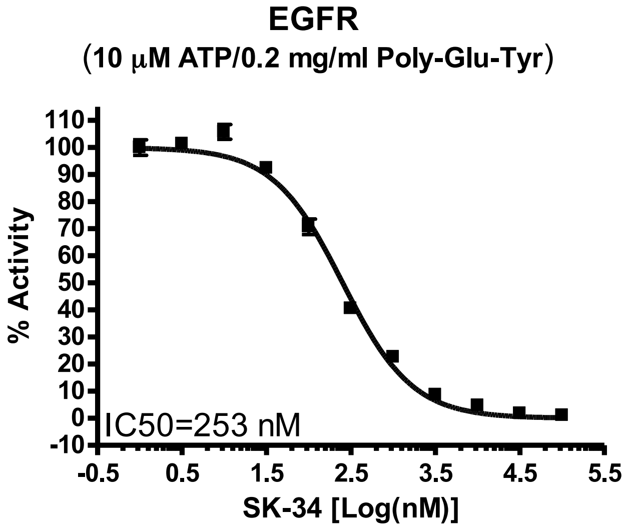

**Table S2: Data for the Effect of SK-18 (compound 2) on EGFR Activity**

| SK-18 (Log [nM]) | EGFR Activity Luminescence | | | % Activity | | |
|---|---|---|---|---|---|---|
| | Set1 | Set2 | Set3 | Set1 | Set2 | Set3 |
| No Compound | 8247 | 8359 | 8110 | 100 | 101 | 98 |
| 0 | 3604 | 3492 | 3444 | 43 | 41 | 41 |
| 1.0 | 2483 | 2486 | 2407 | 29 | 29 | 28 |
| 1.5 | 1042 | 888 | 1009 | 11 | 9 | 11 |
| 2.0 | 595 | 576 | 606 | 6 | 6 | 6 |
| 2.5 | 363 | 316 | 330 | 3 | 2 | 3 |
| 3.0 | 235 | 217 | 240 | 1 | 1 | 1 |
| 3.5 | 184 | 183 | 161 | 1 | 1 | 0 |
| 4.0 | 135 | 130 | 143 | 0 | 0 | 0 |
| 4.5 | 149 | 133 | 112 | 0 | 0 | 0 |
| 5.0 | 123 | 164 | 128 | 0 | 0 | 0 |
| Background | 119 | 134 | 126 | | | |

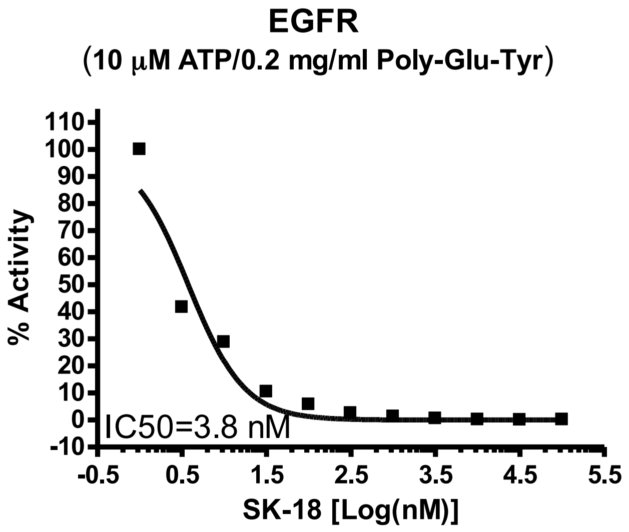

**Table S3: Data for the Effect of SK-17 (compound 3) on EGFR Activity**

| SK-17 (Log [nM]) | EGFR Activity Luminescence | | | % Activity | | |
|---|---|---|---|---|---|---|
| | Set1 | Set2 | Set3 | Set1 | Set2 | Set3 |
| No Compound | 8572 | 8464 | 7927 | 103 | 102 | 95 |
| 0 | 4808 | 4889 | 4706 | 57 | 58 | 56 |
| 1.0 | 3202 | 3158 | 3419 | 38 | 37 | 40 |
| 1.5 | 1210 | 1432 | 1434 | 13 | 16 | 16 |
| 2.0 | 695 | 683 | 781 | 7 | 7 | 8 |
| 2.5 | 328 | 416 | 405 | 2 | 4 | 3 |
| 3.0 | 231 | 245 | 260 | 1 | 1 | 2 |
| 3.5 | 162 | 185 | 165 | 0 | 1 | 0 |
| 4.0 | 136 | 153 | 143 | 0 | 0 | 0 |
| 4.5 | 133 | 131 | 131 | 0 | 0 | 0 |
| 5.0 | 132 | 141 | 128 | 0 | 0 | 0 |
| Background | 127 | 116 | 132 | | | |

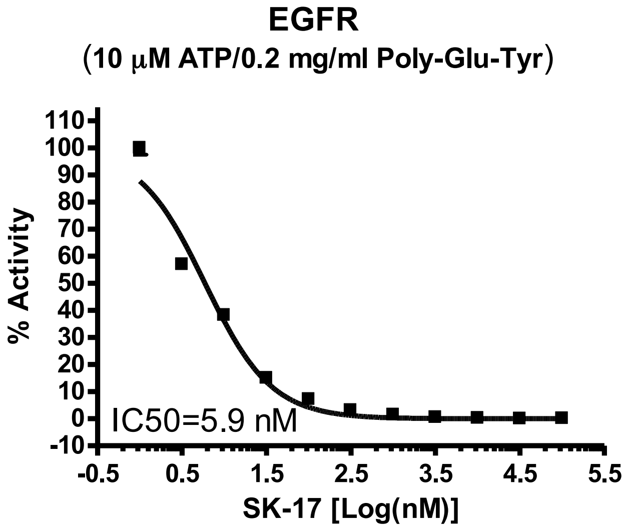

**Table S4: Data for the Effect of TM-9 (compound 4) on EGFR Activity**

| TM-9 (Log [nM]) | EGFR Activity Luminescence | | | % Activity | | |
|---|---|---|---|---|---|---|
| | Set1 | Set2 | Set3 | Set1 | Set2 | Set3 |
| No Compound | 7106 | 7597 | 7685 | 95 | 102 | 103 |
| 0 | 7599 | 7194 | 7272 | 102 | 96 | 97 |
| 1.0 | 7608 | 7225 | 7193 | 102 | 97 | 96 |
| 1.5 | 4897 | 4575 | 4833 | 65 | 61 | 64 |
| 2.0 | 3785 | 3448 | 3952 | 50 | 45 | 52 |
| 2.5 | 1550 | 1517 | 1712 | 19 | 19 | 22 |
| 3.0 | 888 | 869 | 975 | 10 | 10 | 12 |
| 3.5 | 403 | 433 | 440 | 4 | 4 | 4 |
| 4.0 | 254 | 257 | 260 | 2 | 2 | 2 |
| 4.5 | 184 | 182 | 163 | 1 | 1 | 1 |
| 5.0 | 159 | 175 | 179 | 1 | 1 | 1 |
| Background | 107 | 135 | 117 | | | |

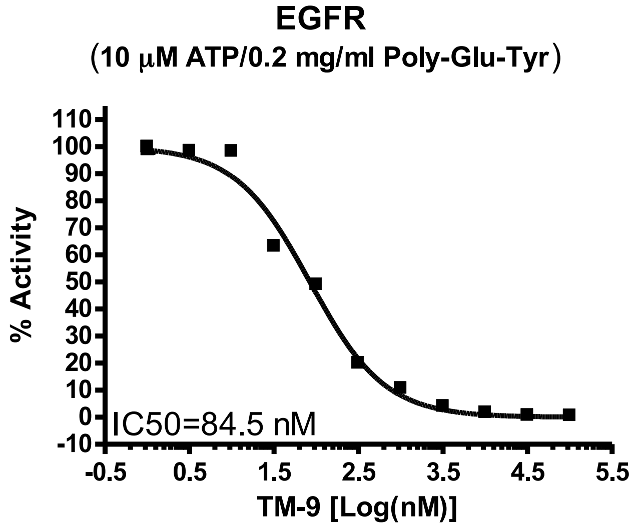

**Table S5: Data for the Effect of SK-32 (compound 5) on Kinase Activity**

| SK-32 (Log [nM]) | EGFR Activity Luminescence | | | % Activity | | |
|---|---|---|---|---|---|---|
| | Set1 | Set2 | Set3 | Set1 | Set2 | Set3 |
| No Compound | 7168 | 7732 | 7684 | 95 | 103 | 102 |
| 0 | 3874 | 4474 | 4386 | 51 | 59 | 58 |
| 1.0 | 3328 | 3535 | 3663 | 43 | 46 | 48 |
| 1.5 | 1189 | 1284 | 1269 | 14 | 16 | 16 |
| 2.0 | 714 | 768 | 780 | 8 | 9 | 9 |
| 2.5 | 359 | 398 | 459 | 3 | 4 | 5 |
| 3.0 | 235 | 253 | 242 | 2 | 2 | 2 |
| 3.5 | 163 | 165 | 165 | 1 | 1 | 1 |
| 4.0 | 123 | 131 | 213 | 0 | 0 | 1 |
| 4.5 | 125 | 133 | 138 | 0 | 0 | 0 |
| 5.0 | 122 | 313 | 158 | 0 | 3 | 1 |
| Background | 114 | 119 | 111 | | | |

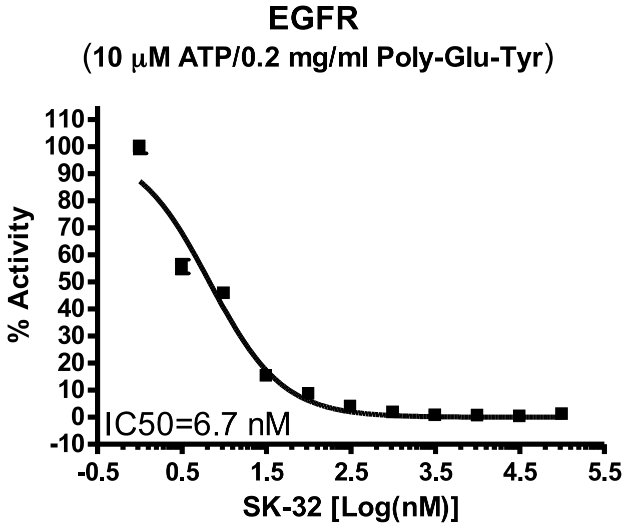

**Table S6: Data for the Effect of BM-7 (compound 6) on EGFR Kinase Activity**

| BM-7 (Log [nM]) | EGFR Activity Luminescence | | | % Activity | | |
|---|---|---|---|---|---|---|
| | Set1 | Set2 | Set3 | Set1 | Set2 | Set3 |
| No Compound | 7916 | 8475 | 7946 | 98 | 105 | 98 |
| 0 | 7931 | 8294 | 8548 | 98 | 102 | 105 |
| 1.0 | 8243 | 8911 | 8738 | 102 | 110 | 108 |
| 1.5 | 8479 | 8559 | 8727 | 105 | 106 | 108 |
| 2.0 | 8059 | 8258 | 8420 | 99 | 102 | 104 |
| 2.5 | 7826 | 7572 | 7833 | 96 | 93 | 97 |
| 3.0 | 5845 | 6420 | 6359 | 72 | 79 | 78 |
| 3.5 | 3535 | 3967 | 3561 | 43 | 48 | 43 |
| 4.0 | 2000 | 2074 | 1912 | 23 | 24 | 22 |
| 4.5 | 783 | 857 | 774 | 8 | 9 | 8 |
| 5.0 | 439 | 448 | 403 | 4 | 4 | 4 |
| Background | 115 | 137 | 117 | | | |

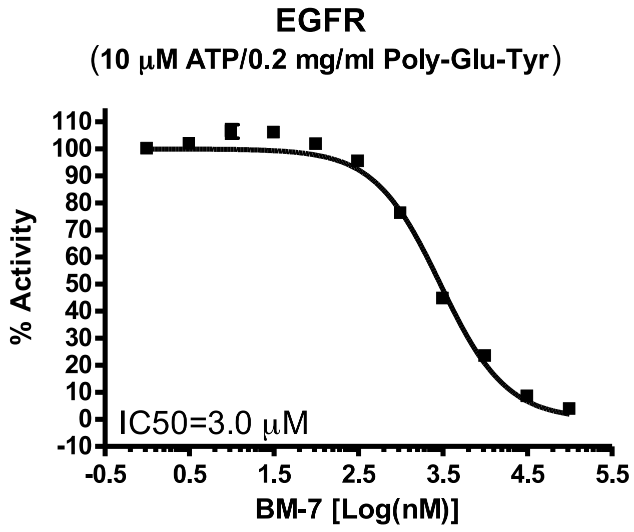

**Table S7: Data for the Effect of BM-19 (compound 7) on EGFR Activity**

| BM-19 (Log [nM]) | EGFR Activity Luminescence | | | % Activity | | |
|---|---|---|---|---|---|---|
| | Set1 | Set2 | Set3 | Set1 | Set2 | Set3 |
| No Compound | 7606 | 7242 | 7558 | 102 | 97 | 101 |
| 0 | 3323 | 3805 | 4016 | 44 | 50 | 53 |
| 1.0 | 1415 | 1335 | 1613 | 18 | 17 | 20 |
| 1.5 | 1013 | 1097 | 1223 | 12 | 13 | 15 |
| 2.0 | 418 | 467 | 440 | 4 | 5 | 4 |
| 2.5 | 347 | 316 | 370 | 3 | 3 | 3 |
| 3.0 | 199 | 247 | 272 | 1 | 2 | 2 |
| 3.5 | 156 | 163 | 148 | 1 | 1 | 0 |
| 4.0 | 136 | 143 | 123 | 0 | 0 | 0 |
| 4.5 | 186 | 176 | 177 | 1 | 1 | 1 |
| 5.0 | 148 | 135 | 132 | 0 | 0 | 0 |
| Background | 125 | 110 | 117 | | | |

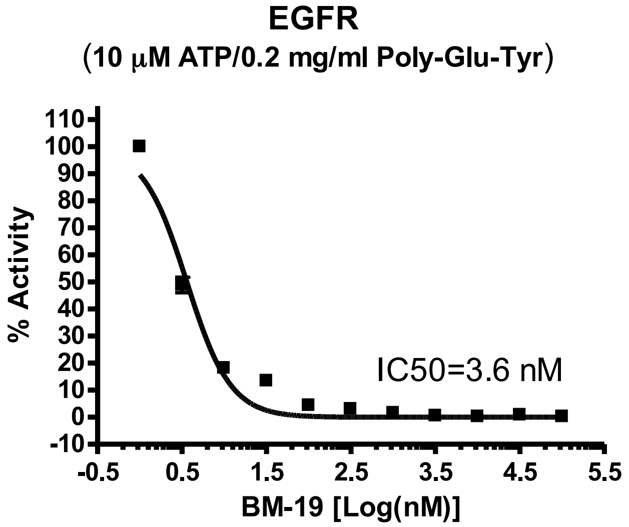

**Table S8: Data for the Effect of AF-36 (compound 8) on EGFR Activity**

| AF-36 (Log [nM]) | EGFR Activity Luminescence | | | % Activity | | |
|---|---|---|---|---|---|---|
| | Set1 | Set2 | Set3 | Set1 | Set2 | Set3 |
| No Compound | 9729 | 10039 | 9851 | 98 | 102 | 100 |
| 0 | 9567 | 9812 | 9790 | 97 | 99 | 99 |
| 1.0 | 9381 | 10015 | 9043 | 95 | 102 | 91 |
| 1.5 | 9236 | 10085 | 8383 | 93 | 102 | 84 |
| 2.0 | 8064 | 8296 | 8177 | 80 | 83 | 82 |
| 2.5 | 5401 | 5165 | 5502 | 51 | 49 | 53 |
| 3.0 | 3054 | 2830 | 3160 | 26 | 23 | 27 |
| 3.5 | 1728 | 1521 | 1591 | 12 | 9 | 10 |
| 4.0 | 1075 | 1135 | 1002 | 4 | 5 | 4 |
| 4.5 | 867 | 946 | 738 | 2 | 3 | 1 |
| 5.0 | 778 | 736 | 811 | 1 | 1 | 2 |
| Background | 702 | 675 | 623 | | | |

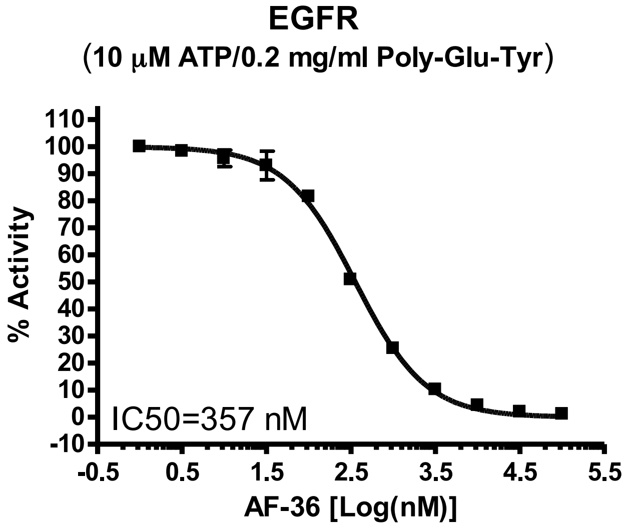

**Table S9: Data for the Effect of BM-26 (compound 9) on Kinase Activity**

| BM-26 (Log [nM]) | EGFR Activity Luminescence | | | % Activity | | |
|---|---|---|---|---|---|---|
| | Set1 | Set2 | Set3 | Set1 | Set2 | Set3 |
| No Compound | 8642 | 8755 | 9597 | 96 | 97 | 107 |
| 0 | 9265 | 8849 | 9375 | 103 | 98 | 105 |
| 1.0 | 10035 | 9759 | 9267 | 112 | 109 | 103 |
| 1.5 | 10113 | 9637 | 8565 | 113 | 108 | 95 |
| 2.0 | 8606 | 7989 | 7398 | 95 | 88 | 81 |
| 2.5 | 6831 | 5932 | 6251 | 74 | 63 | 67 |
| 3.0 | 3701 | 3604 | 4223 | 36 | 35 | 43 |
| 3.5 | 2166 | 2026 | 2618 | 18 | 16 | 23 |
| 4.0 | 1356 | 1259 | 1526 | 8 | 7 | 10 |
| 4.5 | 911 | 982 | 883 | 3 | 4 | 3 |
| 5.0 | 779 | 791 | 814 | 1 | 1 | 2 |
| Background | 609 | 681 | 715 | | | |

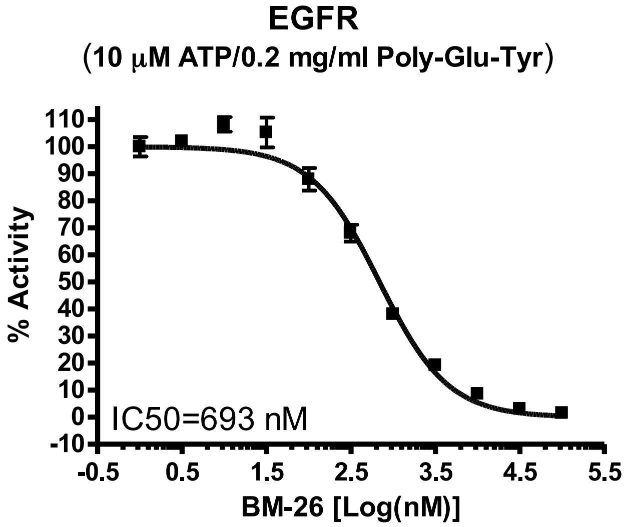

**Table S10: Data for the Effect of BM-22 (compound 10) on Kinase Activity**

| BM-22 (Log [nM]) | EGFR Activity Luminescence | | | % Activity | | |
|---|---|---|---|---|---|---|
| | Set1 | Set2 | Set3 | Set1 | Set2 | Set3 |
| No Compound | 8406 | 7924 | 8198 | 103 | 97 | 100 |
| 0 | 7381 | 7890 | 8454 | 90 | 96 | 103 |
| 1.0 | 7929 | 7395 | 7934 | 97 | 90 | 97 |
| 1.5 | 6377 | 6701 | 6538 | 78 | 82 | 80 |
| 2.0 | 4398 | 4664 | 4222 | 53 | 56 | 51 |
| 2.5 | 2560 | 2486 | 2394 | 30 | 29 | 28 |
| 3.0 | 1237 | 1082 | 1109 | 14 | 12 | 12 |
| 3.5 | 582 | 548 | 539 | 6 | 5 | 5 |
| 4.0 | 303 | 305 | 314 | 2 | 2 | 2 |
| 4.5 | 244 | 242 | 203 | 1 | 1 | 1 |
| 5.0 | 225 | 217 | 220 | 1 | 1 | 1 |
| Background | 123 | 115 | 147 | | | |

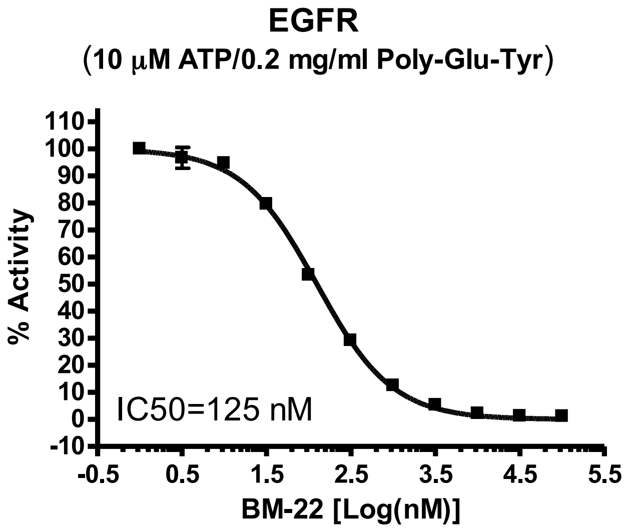

**Table S11: Data for the Effect of BM-31 (compound 11) on Kinase Activity**

| BM-31 (Log [nM]) | EGFR Activity Luminescence | | | % Activity | | |
|---|---|---|---|---|---|---|
| | Set1 | Set2 | Set3 | Set1 | Set2 | Set3 |
| No Compound | 10193 | 9755 | 9586 | 104 | 99 | 97 |
| 0 | 6714 | 6349 | 6470 | 66 | 62 | 63 |
| 1.0 | 3992 | 3767 | 4275 | 36 | 34 | 39 |
| 1.5 | 2259 | 2151 | 2492 | 17 | 16 | 20 |
| 2.0 | 1419 | 1332 | 1727 | 8 | 7 | 11 |
| 2.5 | 1227 | 1057 | 1195 | 6 | 4 | 6 |
| 3.0 | 965 | 736 | 905 | 3 | 1 | 2 |
| 3.5 | 889 | 688 | 678 | 2 | 0 | 0 |
| 4.0 | 740 | 608 | 619 | 1 | 0 | 0 |
| 4.5 | 728 | 615 | 643 | 0 | 0 | 0 |
| 5.0 | 708 | 708 | 566 | 0 | 0 | 0 |
| Background | 737 | 705 | 605 | | | |

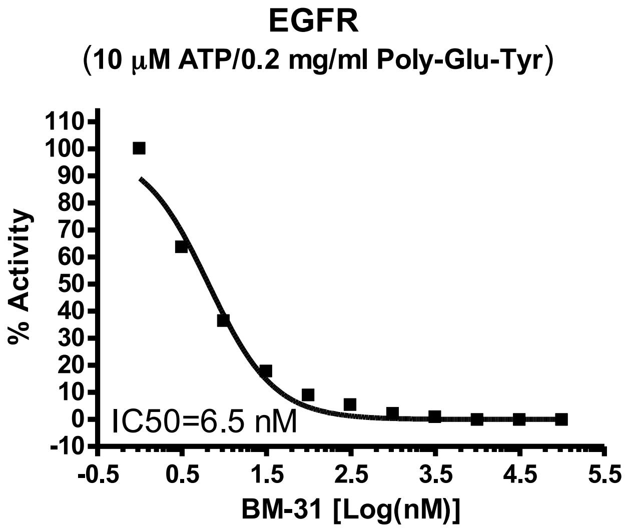

**Table S12: Data for the Effect of BM-25 (compound 12) on Kinase Activity**

| BM-25 (Log [nM]) | EGFR Activity Luminescence | | | % Activity | | |
|---|---|---|---|---|---|---|
| | Set1 | Set2 | Set3 | Set1 | Set2 | Set3 |
| No Compound | 7404 | 7610 | 7571 | 98 | 101 | 101 |
| 0 | 5844 | 6675 | 5735 | 77 | 88 | 76 |
| 1.0 | 5246 | 5682 | 5046 | 69 | 75 | 66 |
| 1.5 | 2654 | 3010 | 2535 | 34 | 39 | 33 |
| 2.0 | 1529 | 1551 | 1306 | 19 | 19 | 16 |
| 2.5 | 846 | 766 | 685 | 10 | 9 | 8 |
| 3.0 | 520 | 496 | 462 | 5 | 5 | 5 |
| 3.5 | 280 | 784 | 271 | 2 | 9 | 2 |
| 4.0 | 174 | 185 | 188 | 1 | 1 | 1 |
| 4.5 | 147 | 155 | 153 | 0 | 0 | 0 |
| 5.0 | 117 | 138 | 134 | 0 | 0 | 0 |
| Background | 123 | 129 | 130 | | | |

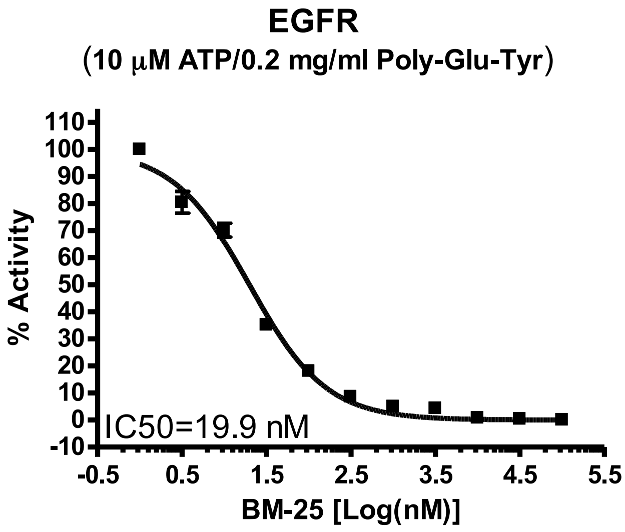

**Table S13: Data for the Effect of BM-34 (compound 13) on Kinase Activity**

| BM-34 (Log [nM]) | EGFR Activity Luminescence | | | % Activity | | |
|---|---|---|---|---|---|---|
| | Set1 | Set2 | Set3 | Set1 | Set2 | Set3 |
| No Compound | 10673 | 11373 | 10570 | 98 | 105 | 97 |
| 0 | 10005 | 10067 | 9924 | 91 | 92 | 91 |
| 1.0 | 7423 | 8429 | 10354 | 66 | 76 | 95 |
| 1.5 | 6162 | 6962 | 6386 | 54 | 61 | 56 |
| 2.0 | 3683 | 3759 | 3931 | 29 | 30 | 32 |
| 2.5 | 2458 | 2668 | 2250 | 17 | 19 | 15 |
| 3.0 | 1558 | 1505 | 1377 | 8 | 8 | 6 |
| 3.5 | 991 | 1087 | 1035 | 3 | 4 | 3 |
| 4.0 | 749 | 857 | 839 | 0 | 1 | 1 |
| 4.5 | 675 | 866 | 766 | 0 | 1 | 0 |
| 5.0 | 714 | 714 | 801 | 0 | 0 | 1 |
| Background | 691 | 681 | 788 | | | |

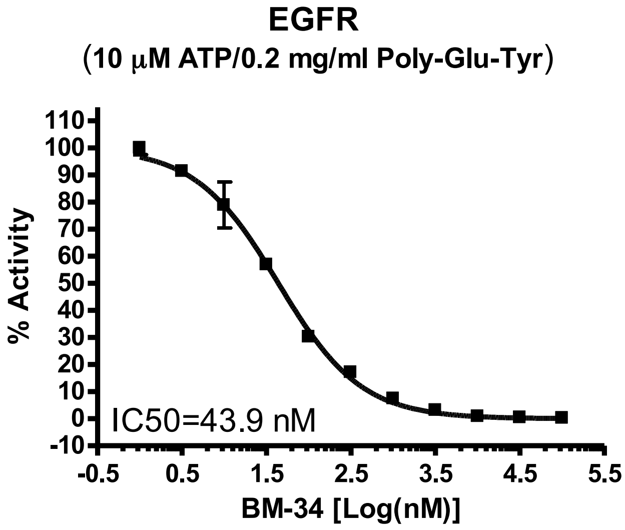

**Table S14: Data for the Effect of BM-17 (compound 14) on Kinase Activity**

| BM-17 (Log [nM]) | EGFR Activity Luminescence | | | % Activity | | |
|---|---|---|---|---|---|---|
| | Set1 | Set2 | Set3 | Set1 | Set2 | Set3 |
| No Compound | 8769 | 8304 | 7968 | 105 | 99 | 95 |
| 0 | 8375 | 7949 | 7887 | 100 | 95 | 94 |
| 1.0 | 8774 | 7492 | 8005 | 105 | 90 | 96 |
| 1.5 | 9013 | 6975 | 7766 | 108 | 83 | 93 |
| 2.0 | 7470 | 6322 | 7043 | 89 | 75 | 84 |
| 2.5 | 5272 | 4502 | 4827 | 62 | 53 | 57 |
| 3.0 | 3081 | 2364 | 2846 | 36 | 27 | 33 |
| 3.5 | 1463 | 1323 | 1426 | 16 | 14 | 16 |
| 4.0 | 830 | 670 | 686 | 8 | 6 | 7 |
| 4.5 | 451 | 373 | 389 | 4 | 3 | 3 |
| 5.0 | 550 | 473 | 489 | 5 | 4 | 4 |
| Background | 170 | 143 | 131 | | | |

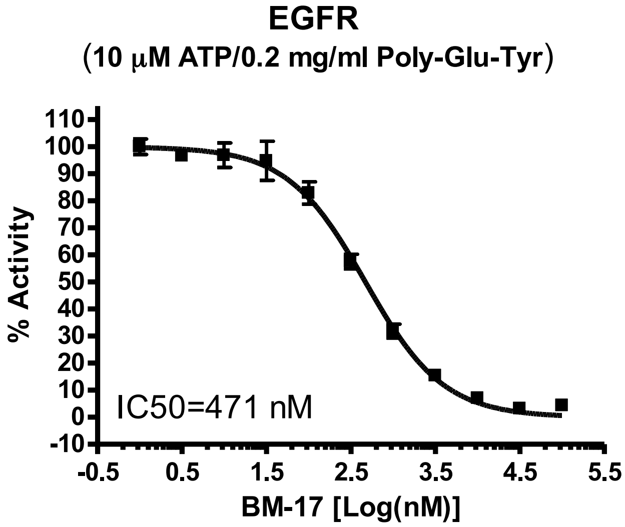

**Table S15: Data for the Effect of BM-24 (compound 15) on Kinase Activity**

| BM-24 (Log [nM]) | EGFR Activity Luminescence | | | % Activity | | |
|---|---|---|---|---|---|---|
| | Set1 | Set2 | Set3 | Set1 | Set2 | Set3 |
| No Compound | 8471 | 9578 | 9891 | 90 | 103 | 107 |
| 0 | 9075 | 9098 | 11146 | 97 | 98 | 121 |
| 1.0 | 10080 | 9442 | 11720 | 109 | 101 | 128 |
| 1.5 | 10467 | 10011 | 11258 | 113 | 108 | 122 |
| 2.0 | 10268 | 9446 | 9212 | 111 | 102 | 99 |
| 2.5 | 7351 | 7756 | 7961 | 77 | 82 | 84 |
| 3.0 | 5432 | 5760 | 5158 | 55 | 59 | 52 |
| 3.5 | 2430 | 2768 | 2653 | 20 | 24 | 23 |
| 4.0 | 1672 | 1714 | 1547 | 12 | 12 | 10 |
| 4.5 | 1014 | 1114 | 1007 | 4 | 5 | 4 |
| 5.0 | 965 | 880 | 926 | 4 | 3 | 3 |
| Background | 655 | 684 | 647 | | | |

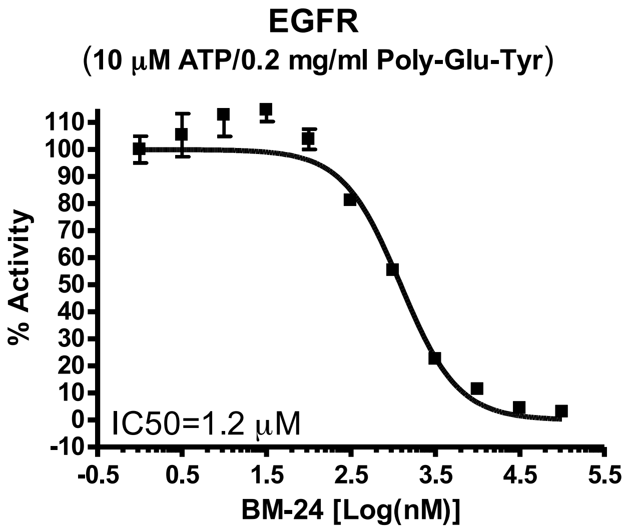

**Table S16: Data for the Effect of RS-48 (compound 16) on Kinase Activity**

| RS-48 (Log [nM]) | EGFR Activity Luminescence | | | % Activity | | |
|---|---|---|---|---|---|---|
| | Set1 | Set2 | Set3 | Set1 | Set2 | Set3 |
| No Compound | 10446 | 10163 | 10100 | 102 | 99 | 99 |
| 0 | 10214 | 10492 | 10039 | 100 | 103 | 98 |
| 1.0 | 11377 | 10864 | 11095 | 111 | 106 | 109 |
| 1.5 | 11017 | 11768 | 10619 | 108 | 115 | 104 |
| 2.0 | 10837 | 10740 | 10951 | 106 | 105 | 107 |
| 2.5 | 10956 | 11243 | 11620 | 107 | 110 | 114 |
| 3.0 | 10698 | 10239 | 11292 | 105 | 100 | 111 |
| 3.5 | 8965 | 9550 | 9871 | 87 | 93 | 96 |
| 4.0 | 7535 | 7482 | 7669 | 73 | 73 | 74 |
| 4.5 | 8344 | 8648 | 9574 | 81 | 84 | 93 |
| 5.0 | 8127 | 8719 | 9104 | 79 | 85 | 89 |
| Background | 185 | 179 | 212 | | | |

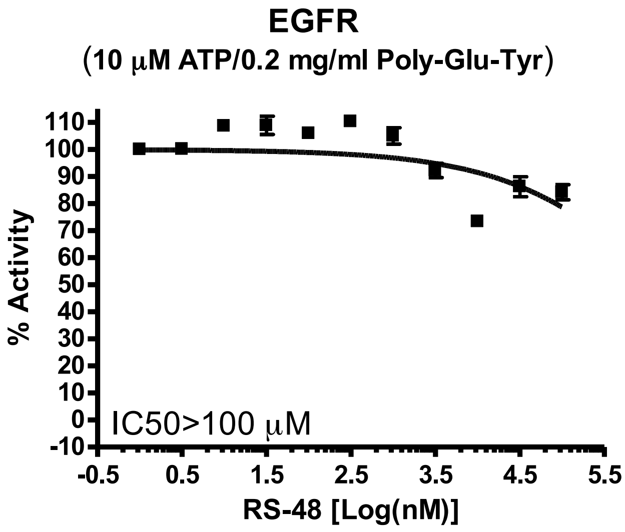

**Table S17: Data for the Effect of BM-23 (compound 17) on Kinase Activity**

| BM-23 (Log [nM]) | EGFR Activity Luminescence | | | % Activity | | |
|---|---|---|---|---|---|---|
| | Set1 | Set2 | Set3 | Set1 | Set2 | Set3 |
| No Compound | 6679 | 7527 | 7688 | 91 | 103 | 105 |
| 0 | 6787 | 7360 | 7260 | 93 | 101 | 99 |
| 1.0 | 6567 | 6682 | 7107 | 90 | 91 | 97 |
| 1.5 | 5290 | 5651 | 6106 | 72 | 77 | 83 |
| 2.0 | 3648 | 3745 | 4072 | 49 | 51 | 55 |
| 2.5 | 1848 | 1747 | 2214 | 24 | 23 | 29 |
| 3.0 | 907 | 810 | 983 | 11 | 10 | 12 |
| 3.5 | 477 | 469 | 511 | 5 | 5 | 5 |
| 4.0 | 406 | 406 | 455 | 4 | 4 | 5 |
| 4.5 | 463 | 472 | 470 | 5 | 5 | 5 |
| 5.0 | 431 | 423 | 429 | 4 | 4 | 4 |
| Background | 119 | 123 | 108 | | | |

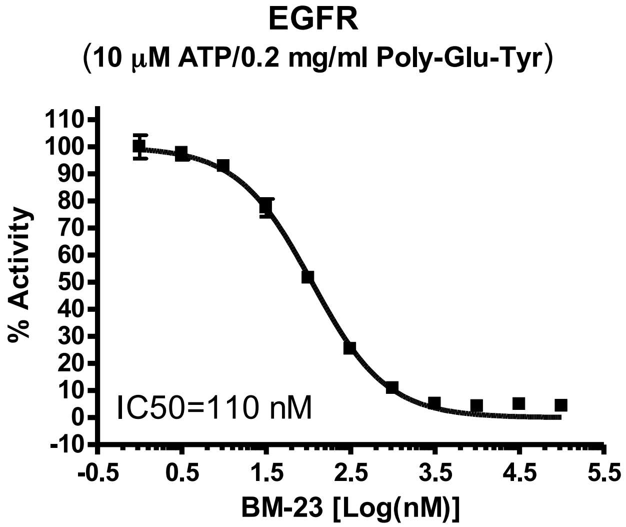

**Table S18: Data for the Effect of SZ-34 (compound 18) on Kinase Activity**

| SZ-34 (Log [nM]) | EGFR Activity Luminescence | | | % Activity | | |
|---|---|---|---|---|---|---|
| | Set1 | Set2 | Set3 | Set1 | Set2 | Set3 |
| No Compound | 9812 | 9614 | 10158 | 99 | 97 | 103 |
| 0 | 9452 | 9109 | 9358 | 96 | 92 | 95 |
| 1.0 | 8721 | 8170 | 8718 | 88 | 83 | 88 |
| 1.5 | 6593 | 6142 | 6443 | 66 | 62 | 65 |
| 2.0 | 4209 | 3862 | 4314 | 42 | 38 | 43 |
| 2.5 | 1767 | 1747 | 1751 | 16 | 16 | 16 |
| 3.0 | 969 | 945 | 1085 | 8 | 8 | 9 |
| 3.5 | 461 | 446 | 482 | 3 | 3 | 3 |
| 4.0 | 378 | 363 | 346 | 2 | 2 | 2 |
| 4.5 | 346 | 350 | 372 | 2 | 2 | 2 |
| 5.0 | 341 | 487 | 337 | 2 | 3 | 2 |
| Background | 189 | 190 | 183 | | | |

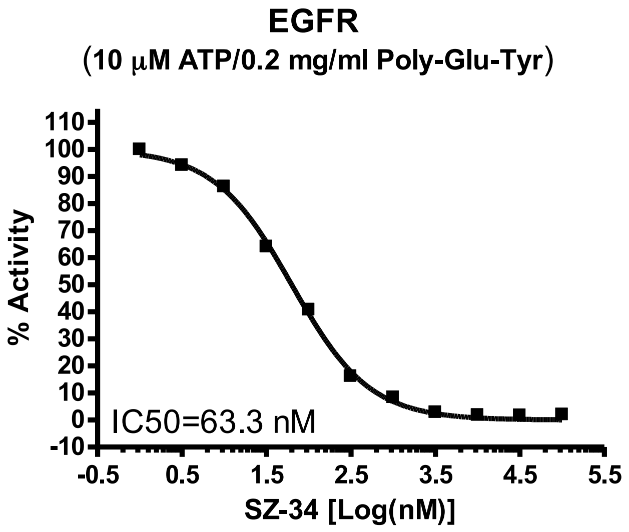

**Table S19: Data for the Effect of Staurosporine on EGFR Activity**

| Staurosporine (Log [nM]) | EGFR Activity Luminescence | | | % Activity | | |
|---|---|---|---|---|---|---|
| | Set1 | Set2 | Set3 | Set1 | Set2 | Set3 |
| No Compound | 8140 | 8688 | 8620 | 96 | 102 | 102 |
| -1.0 | 7563 | 7968 | 8284 | 89 | 94 | 98 |
| -0.5 | 8086 | 8558 | 8505 | 95 | 101 | 100 |
| 0 | 7867 | 8330 | 8179 | 93 | 98 | 96 |
| 1.0 | 6484 | 6960 | 7653 | 76 | 82 | 90 |
| 1.5 | 4694 | 5232 | 5158 | 54 | 61 | 60 |
| 2.0 | 2353 | 2450 | 2652 | 26 | 27 | 30 |
| 2.5 | 1134 | 1036 | 1137 | 12 | 10 | 12 |
| 3.0 | 573 | 642 | 620 | 5 | 6 | 5 |
| 3.5 | 329 | 345 | 366 | 2 | 2 | 2 |
| 4.0 | 249 | 247 | 243 | 1 | 1 | 1 |
| Background | 136 | 149 | 236 | | | |

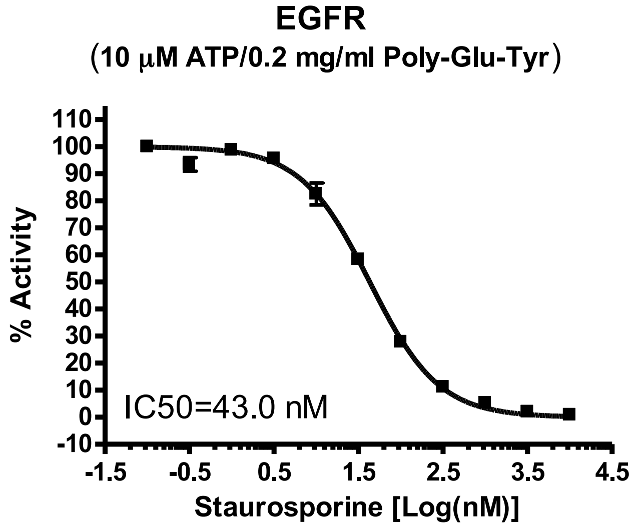

**Table S20: Data for the Effect of SK-34 (compound 1) on AURKA Activity**

| SK-34 (Log [nM]) | AuroraA Activity Luminescence | | | % Activity | | |
|---|---|---|---|---|---|---|
| | Set1 | Set2 | Set3 | Set1 | Set2 | Set3 |
| No Compound | 5578 | 5602 | 5577 | 100 | 100 | 100 |
| 0 | 5068 | 5094 | 5136 | 91 | 91 | 92 |
| 1.0 | 4849 | 5043 | 5114 | 87 | 90 | 91 |
| 1.5 | 5217 | 5477 | 5611 | 93 | 98 | 100 |
| 2.0 | 5357 | 5398 | 5607 | 96 | 97 | 100 |
| 2.5 | 5516 | 5724 | 5806 | 99 | 103 | 104 |
| 3.0 | 4800 | 5043 | 5211 | 86 | 90 | 93 |
| 3.5 | 3538 | 3942 | 3940 | 63 | 70 | 70 |
| 4.0 | 1654 | 1787 | 1871 | 28 | 31 | 32 |
| 4.5 | 717 | 773 | 796 | 11 | 12 | 13 |
| 5.0 | 347 | 395 | 394 | 5 | 5 | 5 |
| Background | 89 | 94 | 104 | | | |

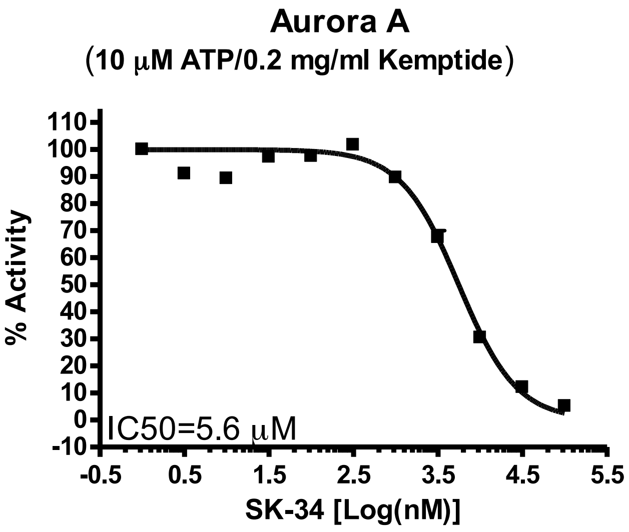

**Table S21: Data for the Effect of SK-18 (compound 2) on AURKA Activity**

| SK-18 (Log [nM]) | AuroraA Activity Luminescence | | | % Activity | | |
|---|---|---|---|---|---|---|
| | Set1 | Set2 | Set3 | Set1 | Set2 | Set3 |
| No Compound | 5264 | 5042 | 5127 | 102 | 98 | 100 |
| 0 | 5510 | 4820 | 4860 | 107 | 94 | 94 |
| 1.0 | 5603 | 4969 | 4952 | 109 | 97 | 96 |
| 1.5 | 5578 | 4922 | 5203 | 109 | 96 | 101 |
| 2.0 | 5270 | 5013 | 5387 | 102 | 97 | 105 |
| 2.5 | 4854 | 4562 | 4905 | 94 | 88 | 95 |
| 3.0 | 3650 | 3428 | 3600 | 70 | 66 | 69 |
| 3.5 | 1886 | 1812 | 1820 | 35 | 34 | 34 |
| 4.0 | 901 | 826 | 831 | 16 | 14 | 14 |
| 4.5 | 393 | 383 | 384 | 6 | 5 | 5 |
| 5.0 | 366 | 368 | 341 | 5 | 5 | 5 |
| Background | 131 | 94 | 96 | | | |

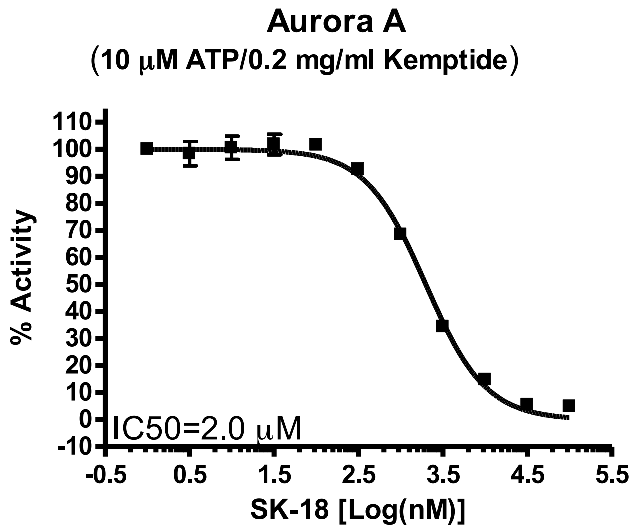

**Table S22: Data for the Effect of SK-17 (compound 3) on AURKA Activity**

| SK-17 (Log [nM]) | AuroraA Activity Luminescence | | | % Activity | | |
|---|---|---|---|---|---|---|
| | Set1 | Set2 | Set3 | Set1 | Set2 | Set3 |
| No Compound | 3875 | 3936 | 3969 | 99 | 100 | 101 |
| 0 | 3725 | 3643 | 3908 | 95 | 93 | 100 |
| 1.0 | 3884 | 3792 | 3972 | 99 | 96 | 101 |
| 1.5 | 3892 | 3705 | 4112 | 99 | 94 | 105 |
| 2.0 | 4127 | 4093 | 4204 | 105 | 104 | 107 |
| 2.5 | 4077 | 4114 | 4057 | 104 | 105 | 103 |
| 3.0 | 3075 | 2967 | 3034 | 78 | 75 | 77 |
| 3.5 | 2130 | 2260 | 2097 | 53 | 57 | 52 |
| 4.0 | 761 | 714 | 708 | 17 | 16 | 16 |
| 4.5 | 351 | 327 | 353 | 7 | 6 | 7 |
| 5.0 | 322 | 280 | 292 | 6 | 5 | 5 |
| Background | 93 | 93 | 83 | | | |

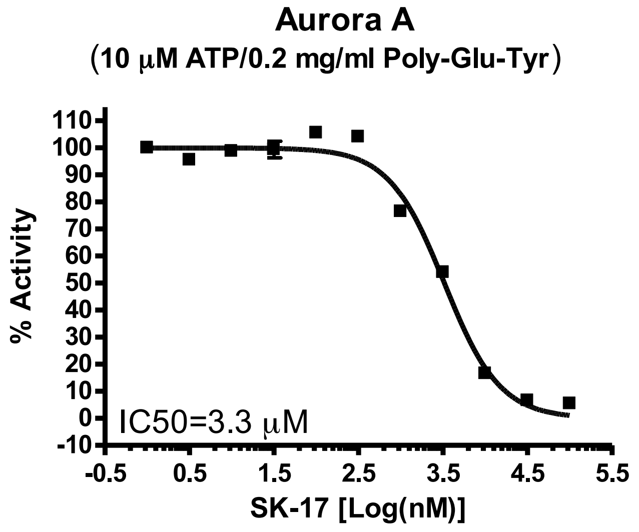

**Table S23: Data for the Effect of TM-9 (compound 4) on AURKA Activity**

| TM-9 (Log [nM]) | AuroraA Activity Luminescence | | | % Activity | | |
|---|---|---|---|---|---|---|
| | Set1 | Set2 | Set3 | Set1 | Set2 | Set3 |
| No Compound | 4310 | 4797 | 4341 | 96 | 107 | 97 |
| 0 | 4387 | 4548 | 4329 | 98 | 101 | 97 |
| 1.0 | 4454 | 4380 | 4502 | 99 | 98 | 100 |
| 1.5 | 4668 | 4832 | 4550 | 104 | 108 | 102 |
| 2.0 | 4697 | 4783 | 4659 | 105 | 107 | 104 |
| 2.5 | 4622 | 4432 | 4506 | 103 | 99 | 101 |
| 3.0 | 3949 | 4169 | 3875 | 88 | 93 | 86 |
| 3.5 | 2972 | 3099 | 3064 | 66 | 69 | 68 |
| 4.0 | 1163 | 1292 | 1202 | 25 | 28 | 25 |
| 4.5 | 580 | 563 | 545 | 11 | 11 | 11 |
| 5.0 | 466 | 501 | 462 | 9 | 10 | 9 |
| Background | 93 | 76 | 76 | | | |

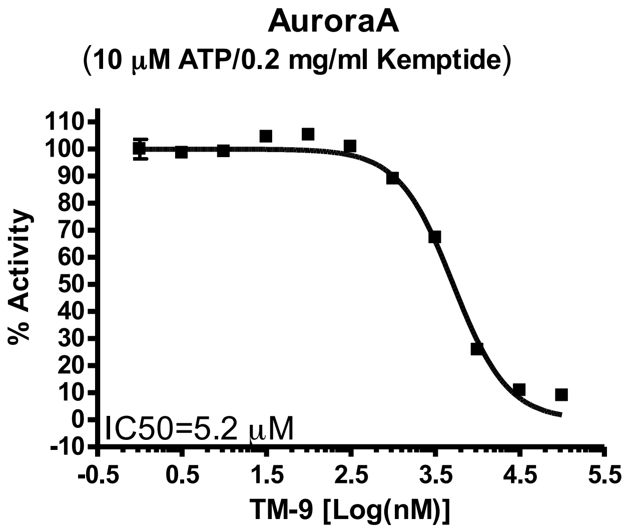

## Table S24: Data for the Effect of SK-32 (compound 5) on AURKA Activity

| SK-32 (Log [nM]) | AuroraA Activity Luminescence | | | % Activity | | |
|---|---|---|---|---|---|---|
| | Set1 | Set2 | Set3 | Set1 | Set2 | Set3 |
| No Compound | 4416 | 4531 | 4800 | 96 | 99 | 105 |
| 0 | 4357 | 4466 | 4728 | 95 | 97 | 103 |
| 1.0 | 4756 | 4350 | 4445 | 104 | 95 | 97 |
| 1.5 | 4514 | 4649 | 4672 | 98 | 101 | 102 |
| 2.0 | 4780 | 4903 | 4795 | 104 | 107 | 105 |
| 2.5 | 4736 | 4921 | 4847 | 103 | 108 | 106 |
| 3.0 | 4166 | 4752 | 4185 | 91 | 104 | 91 |
| 3.5 | 2575 | 2402 | 2681 | 55 | 51 | 58 |
| 4.0 | 808 | 1023 | 942 | 16 | 21 | 19 |
| 4.5 | 580 | 623 | 598 | 11 | 12 | 11 |
| 5.0 | 584 | 612 | 665 | 11 | 12 | 13 |
| Background | 92 | 97 | 81 | | | |

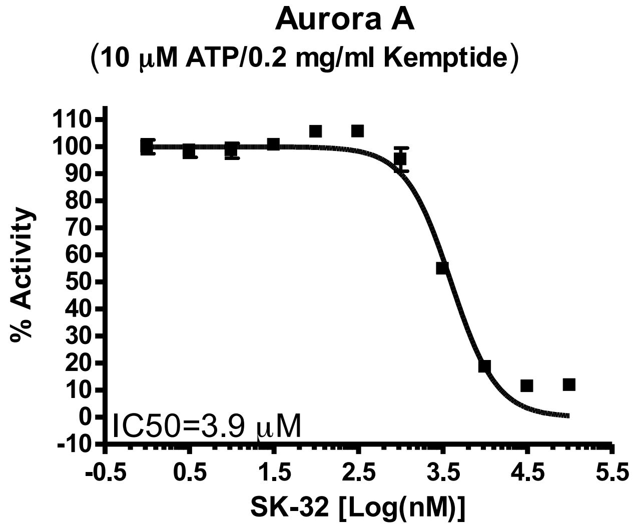

**Table S25: Data for the Effect of BM-7 (compound 6) on AURKA Activity**

| BM-7 (Log [nM]) | AuroraA Luminescence | | | % Activity | | |
|---|---|---|---|---|---|---|
| | Set1 | Set2 | Set3 | Set1 | Set2 | Set3 |
| No Compound | 4334 | 4284 | 4248 | 101 | 100 | 99 |
| 0 | 4436 | 4028 | 3776 | 104 | 94 | 88 |
| 1.0 | 4065 | 3841 | 3893 | 95 | 89 | 91 |
| 1.5 | 4759 | 4272 | 4706 | 111 | 100 | 110 |
| 2.0 | 4308 | 4074 | 4129 | 100 | 95 | 96 |
| 2.5 | 4623 | 4268 | 4520 | 108 | 100 | 106 |
| 3.0 | 3791 | 3909 | 3639 | 88 | 91 | 84 |
| 3.5 | 3723 | 3454 | 3267 | 86 | 80 | 76 |
| 4.0 | 1386 | 1497 | 1254 | 30 | 33 | 27 |
| 4.5 | 708 | 701 | 640 | 14 | 14 | 13 |
| 5.0 | 433 | 348 | 282 | 8 | 6 | 4 |
| Background | 176 | 88 | 76 | | | |

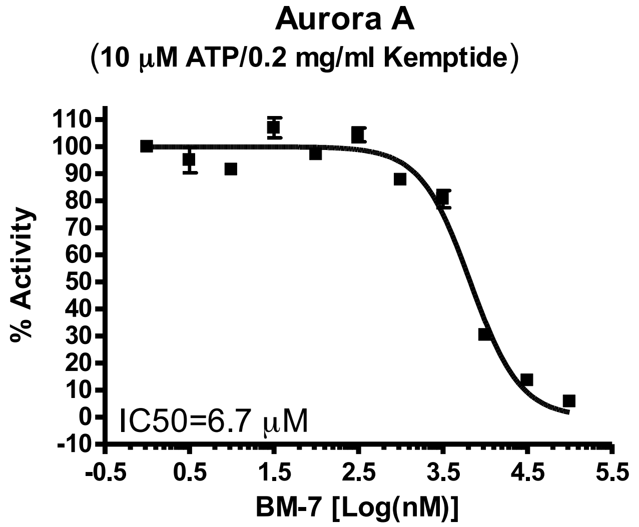

**Table S26: Data for the Effect of BM-19 (compound 7) on AURKA Activity**

| BM-19 (Log [nM]) | AuroraA Activity Luminescence | | | % Activity | | |
|---|---|---|---|---|---|---|
| | Set1 | Set2 | Set3 | Set1 | Set2 | Set3 |
| No Compound | 5013 | 4935 | 5330 | 98 | 97 | 105 |
| 0 | 4855 | 5181 | 4855 | 95 | 102 | 95 |
| 1.0 | 5100 | 5260 | 5047 | 100 | 103 | 99 |
| 1.5 | 5090 | 5239 | 5227 | 100 | 103 | 103 |
| 2.0 | 5094 | 5374 | 5354 | 100 | 106 | 105 |
| 2.5 | 4839 | 4931 | 4845 | 95 | 97 | 95 |
| 3.0 | 4823 | 5237 | 4747 | 95 | 103 | 93 |
| 3.5 | 2097 | 2110 | 1950 | 40 | 40 | 37 |
| 4.0 | 891 | 901 | 837 | 16 | 16 | 15 |
| 4.5 | 1656 | 1607 | 1615 | 31 | 30 | 30 |
| 5.0 | 934 | 854 | 899 | 17 | 15 | 16 |
| Background | 124 | 98 | 100 | | | |

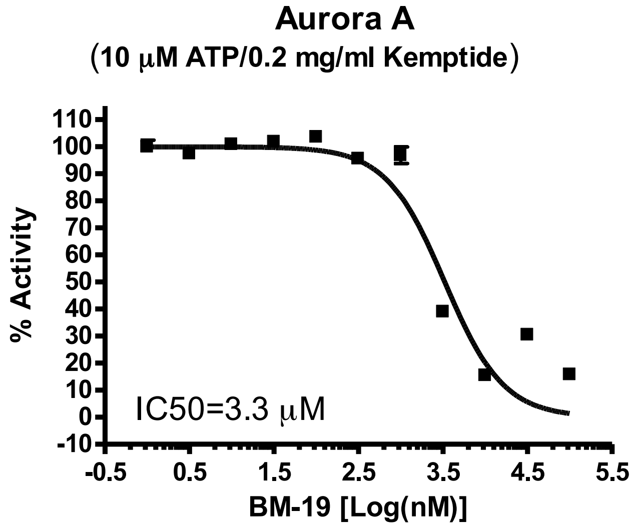

**Table S27: Data for the Effect of AF-36 (compound 8) on AURKA Activity**

| AF-36 (Log [nM]) | Aurora A Luminescence | | | % Activity | | |
|---|---|---|---|---|---|---|
| | Set1 | Set2 | Set3 | Set1 | Set2 | Set3 |
| No Compound | 3685 | 4270 | 3915 | 93 | 108 | 99 |
| 0 | 4065 | 4229 | 3922 | 103 | 107 | 99 |
| 1.0 | 4346 | 3983 | 4371 | 110 | 101 | 111 |
| 1.5 | 4290 | 4254 | 4144 | 109 | 108 | 105 |
| 2.0 | 4297 | 4292 | 4296 | 109 | 109 | 109 |
| 2.5 | 3823 | 3977 | 4464 | 96 | 101 | 113 |
| 3.0 | 3343 | 3214 | 3640 | 84 | 80 | 92 |
| 3.5 | 2232 | 2308 | 2415 | 55 | 57 | 59 |
| 4.0 | 1294 | 1236 | 1256 | 30 | 28 | 29 |
| 4.5 | 556 | 576 | 578 | 11 | 11 | 11 |
| 5.0 | 464 | 429 | 443 | 8 | 7 | 8 |
| Background | 150 | 143 | 163 | | | |

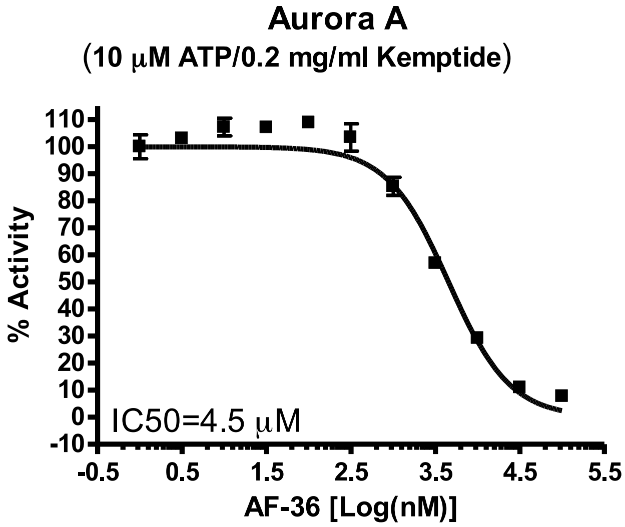

**Table S28: Data for the Effect of BM-26 (compound 9) on Kinase Activity**

| BM-26 (Log [nM]) | Aurora A Luminescence | | | % Activity | | |
|---|---|---|---|---|---|---|
| | Set1 | Set2 | Set3 | Set1 | Set2 | Set3 |
| No Compound | 3868 | 4131 | 3910 | 97 | 104 | 98 |
| 0 | 3999 | 4099 | 4157 | 101 | 103 | 105 |
| 1.0 | 4501 | 4095 | 4228 | 114 | 103 | 107 |
| 1.5 | 4381 | 4093 | 4325 | 111 | 103 | 109 |
| 2.0 | 4050 | 4237 | 4501 | 102 | 107 | 114 |
| 2.5 | 3935 | 4051 | 4369 | 99 | 102 | 110 |
| 3.0 | 3469 | 3595 | 3616 | 87 | 90 | 91 |
| 3.5 | 2541 | 2681 | 2490 | 63 | 66 | 61 |
| 4.0 | 1353 | 1268 | 1299 | 32 | 29 | 30 |
| 4.5 | 747 | 712 | 655 | 16 | 15 | 13 |
| 5.0 | 494 | 478 | 486 | 9 | 9 | 9 |
| Background | 139 | 141 | 135 | | | |

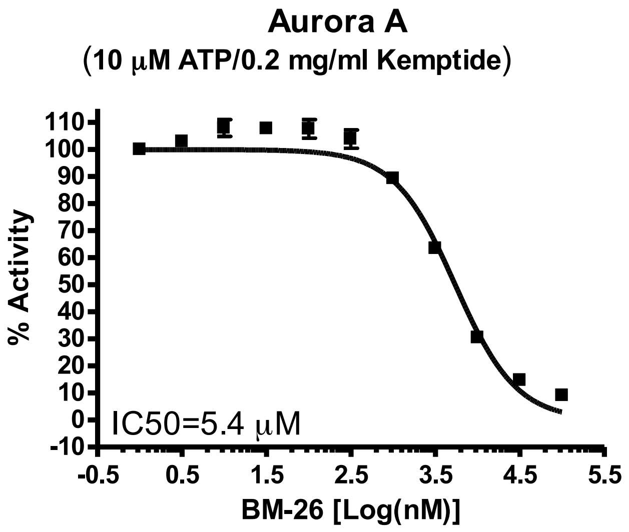

**Table S29: Data for the Effect of BM-22 (compound 10) on Kinase Activity**

| BM-22 (Log [nM]) | AuroraA Activity Luminescence | | | % Activity | | |
|---|---|---|---|---|---|---|
| | Set1 | Set2 | Set3 | Set1 | Set2 | Set3 |
| No Compound | 4934 | 4986 | 4984 | 99 | 100 | 100 |
| 0 | 4857 | 4318 | 4783 | 98 | 87 | 96 |
| 1.0 | 4805 | 4689 | 4691 | 97 | 94 | 94 |
| 1.5 | 4944 | 4709 | 4733 | 100 | 95 | 95 |
| 2.0 | 5056 | 4805 | 5035 | 102 | 97 | 101 |
| 2.5 | 4952 | 4638 | 4862 | 100 | 93 | 98 |
| 3.0 | 4114 | 4141 | 4097 | 82 | 83 | 82 |
| 3.5 | 3037 | 2771 | 2702 | 60 | 55 | 53 |
| 4.0 | 1093 | 1027 | 1090 | 20 | 19 | 20 |
| 4.5 | 521 | 524 | 548 | 9 | 9 | 9 |
| 5.0 | 577 | 587 | 547 | 10 | 10 | 9 |
| Background | 98 | 97 | 102 | | | |

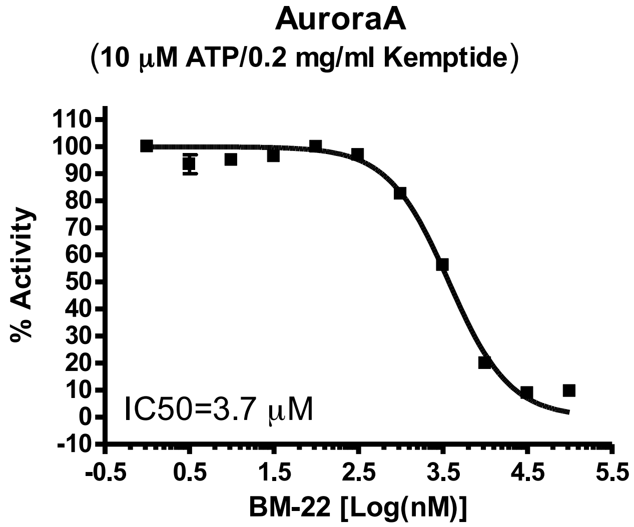

**Table S30: Data for the Effect of BM-31 (compound 11) on Kinase Activity**

| BM-31 (Log [nM]) | Aurora A Luminescence | | | % Activity | | |
|---|---|---|---|---|---|---|
| | Set1 | Set2 | Set3 | Set1 | Set2 | Set3 |
| No Compound | 4407 | 4193 | 3800 | 107 | 102 | 92 |
| 0 | 4081 | 4120 | 3719 | 99 | 100 | 90 |
| 1.0 | 4475 | 3951 | 4167 | 109 | 95 | 101 |
| 1.5 | 4228 | 4442 | 3961 | 102 | 108 | 96 |
| 2.0 | 3908 | 4279 | 4413 | 94 | 104 | 107 |
| 2.5 | 3951 | 4046 | 4079 | 95 | 98 | 99 |
| 3.0 | 3323 | 3521 | 3489 | 80 | 85 | 84 |
| 3.5 | 1838 | 2057 | 2108 | 42 | 48 | 49 |
| 4.0 | 925 | 1013 | 901 | 19 | 22 | 19 |
| 4.5 | 467 | 508 | 456 | 8 | 9 | 8 |
| 5.0 | 353 | 298 | 289 | 5 | 4 | 3 |
| Background | 150 | 163 | 157 | | | |

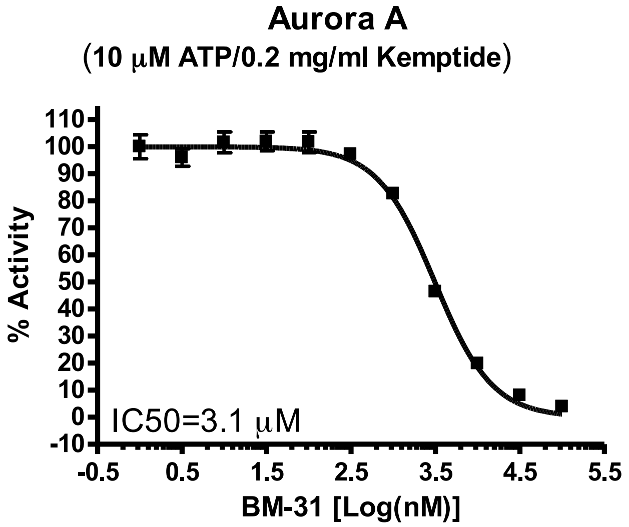

**Table S31: Data for the Effect of BM-25 (compound 12) on Kinase Activity**

| BM-25 (Log [nM]) | AuroraA Activity Luminescence | | | % Activity | | |
|---|---|---|---|---|---|---|
| | Set1 | Set2 | Set3 | Set1 | Set2 | Set3 |
| No Compound | 4617 | 4910 | 4949 | 96 | 102 | 103 |
| 0 | 4881 | 5079 | 4586 | 101 | 105 | 95 |
| 1.0 | 4843 | 4906 | 4688 | 100 | 102 | 97 |
| 1.5 | 4697 | 4844 | 4665 | 97 | 100 | 97 |
| 2.0 | 4638 | 4895 | 5042 | 96 | 101 | 105 |
| 2.5 | 4318 | 4590 | 4604 | 89 | 95 | 95 |
| 3.0 | 4307 | 4382 | 4425 | 89 | 91 | 92 |
| 3.5 | 2388 | 2451 | 2623 | 48 | 50 | 53 |
| 4.0 | 926 | 902 | 904 | 17 | 17 | 17 |
| 4.5 | 453 | 492 | 477 | 7 | 8 | 8 |
| 5.0 | 272 | 277 | 263 | 4 | 4 | 3 |
| Background | 115 | 99 | 85 | | | |

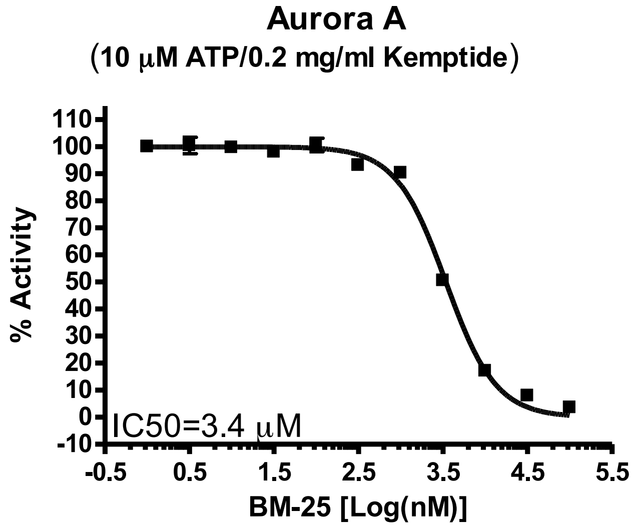

**Table S32: Data for the Effect of BM-34 (compound 13) on Kinase Activity**

| BM-34 (Log [nM]) | Aurora A Luminescence | | | % Activity | | |
|---|---|---|---|---|---|---|
| | Set1 | Set2 | Set3 | Set1 | Set2 | Set3 |
| No Compound | 4440 | 4084 | 4139 | 105 | 97 | 98 |
| 0 | 3989 | 4279 | 4060 | 94 | 101 | 96 |
| 1.0 | 4333 | 4252 | 4457 | 103 | 101 | 106 |
| 1.5 | 4506 | 4747 | 4983 | 107 | 113 | 119 |
| 2.0 | 4631 | 4550 | 4758 | 110 | 108 | 113 |
| 2.5 | 4535 | 4530 | 4726 | 108 | 108 | 112 |
| 3.0 | 3994 | 4240 | 3965 | 94 | 100 | 94 |
| 3.5 | 3132 | 2699 | 3103 | 73 | 63 | 72 |
| 4.0 | 1467 | 1201 | 1297 | 32 | 26 | 28 |
| 4.5 | 697 | 568 | 606 | 13 | 10 | 11 |
| 5.0 | 732 | 618 | 691 | 14 | 11 | 13 |
| Background | 157 | 164 | 153 | | | |

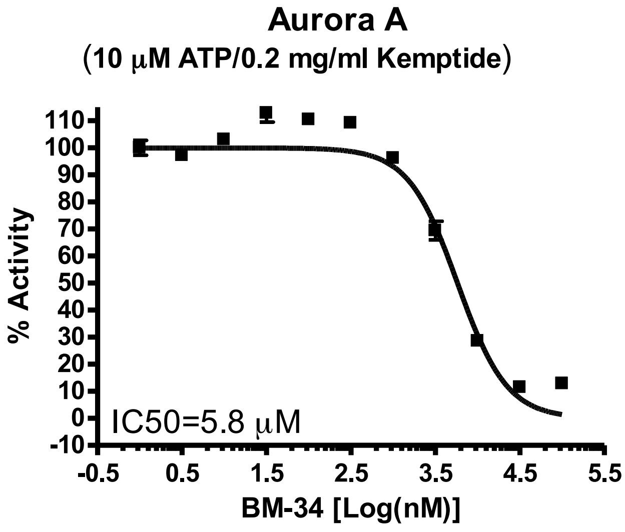

**Table S33: Data for the Effect of BM-17 (compound 14) on Kinase Activity**

| BM-17 (Log [nM]) | AuroraA Activity Luminescence | | | % Activity | | |
|---|---|---|---|---|---|---|
| | Set1 | Set2 | Set3 | Set1 | Set2 | Set3 |
| No Compound | 4898 | 4803 | 4768 | 102 | 100 | 99 |
| 0 | 4682 | 4436 | 4551 | 97 | 92 | 94 |
| 1.0 | 4935 | 4693 | 4706 | 102 | 97 | 98 |
| 1.5 | 5329 | 4628 | 4728 | 111 | 96 | 98 |
| 2.0 | 4963 | 4705 | 4928 | 103 | 98 | 102 |
| 2.5 | 4869 | 5083 | 4873 | 101 | 106 | 101 |
| 3.0 | 4192 | 4158 | 4478 | 87 | 86 | 93 |
| 3.5 | 3529 | 3301 | 3538 | 73 | 68 | 73 |
| 4.0 | 1433 | 1264 | 1381 | 28 | 25 | 27 |
| 4.5 | 755 | 729 | 782 | 14 | 13 | 14 |
| 5.0 | 727 | 636 | 759 | 13 | 11 | 14 |
| Background | 98 | 87 | 107 | | | |

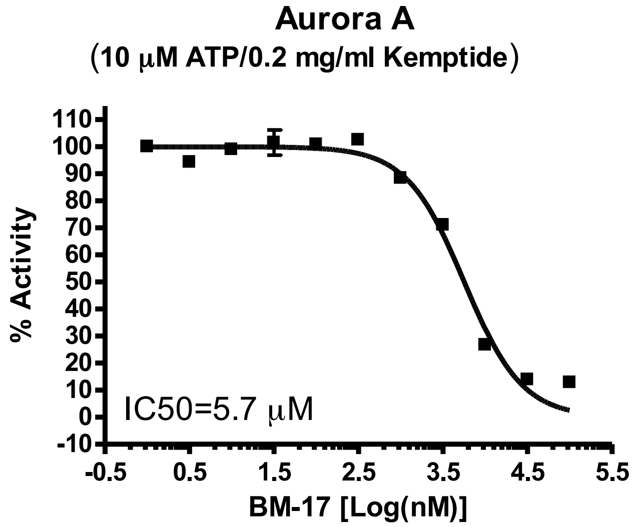

**Table S34: Data for the Effect of BM-24 (compound 15) on Kinase Activity**

| BM-24 (Log [nM]) | Aurora A Luminescence | | | % Activity | | |
|---|---|---|---|---|---|---|
| | Set1 | Set2 | Set3 | Set1 | Set2 | Set3 |
| No Compound | 4370 | 4017 | 4234 | 104 | 95 | 101 |
| 0 | 3984 | 4087 | 4270 | 95 | 97 | 102 |
| 1.0 | 4279 | 4321 | 4304 | 102 | 103 | 102 |
| 1.5 | 4400 | 4424 | 4509 | 105 | 105 | 107 |
| 2.0 | 4134 | 4490 | 4575 | 98 | 107 | 109 |
| 2.5 | 4316 | 4311 | 4496 | 103 | 103 | 107 |
| 3.0 | 3883 | 4284 | 4117 | 92 | 102 | 98 |
| 3.5 | 2811 | 3127 | 2989 | 66 | 73 | 70 |
| 4.0 | 1941 | 2086 | 2077 | 44 | 48 | 48 |
| 4.5 | 876 | 868 | 916 | 18 | 18 | 19 |
| 5.0 | 577 | 577 | 594 | 11 | 11 | 11 |
| Background | 138 | 145 | 151 | | | |

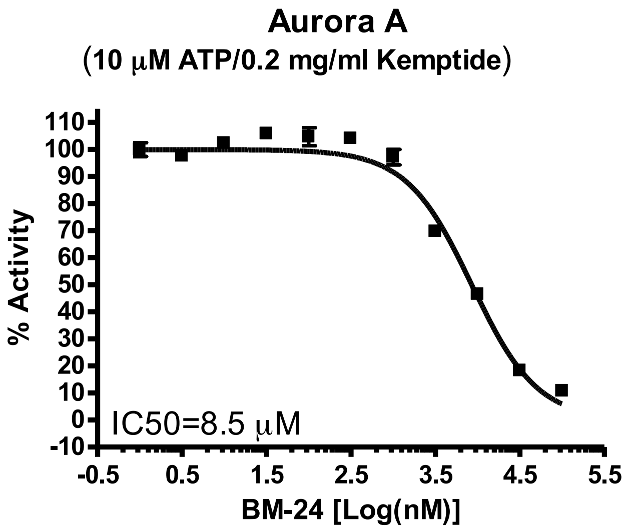

**Table S35: Data for the Effect of RS-48 (compound 16) on Kinase Activity**

| RS-48 (Log [nM]) | Aurora A Luminescence | | | % Activity | | |
|---|---|---|---|---|---|---|
| | Set1 | Set2 | Set3 | Set1 | Set2 | Set3 |
| No Compound | 5639 | 5518 | 5756 | 100 | 98 | 102 |
| 0 | 5322 | 5167 | 5357 | 94 | 91 | 95 |
| 1.0 | 5072 | 5097 | 5543 | 90 | 90 | 98 |
| 1.5 | 4873 | 5105 | 5307 | 86 | 90 | 94 |
| 2.0 | 4807 | 4865 | 5322 | 85 | 86 | 94 |
| 2.5 | 4763 | 4987 | 5286 | 84 | 88 | 94 |
| 3.0 | 4360 | 4787 | 4726 | 77 | 85 | 84 |
| 3.5 | 3307 | 3405 | 3314 | 58 | 60 | 58 |
| 4.0 | 2601 | 2406 | 2630 | 45 | 42 | 46 |
| 4.5 | 3187 | 3466 | 3670 | 56 | 61 | 64 |
| 5.0 | 3167 | 3298 | 3643 | 55 | 58 | 64 |
| Background | 110 | 100 | 103 | | | |

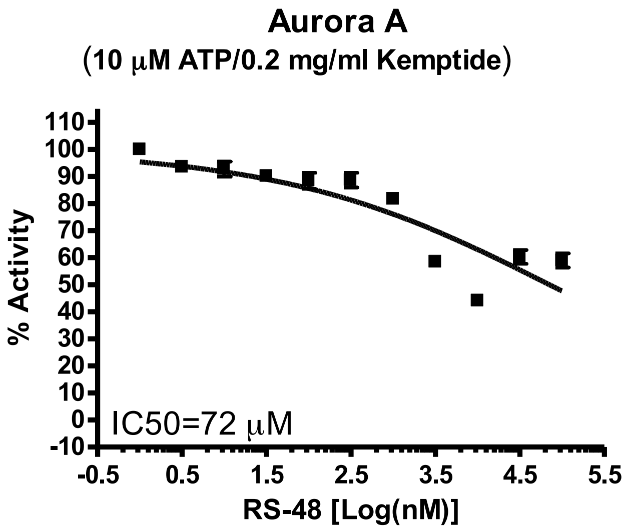

**Table S36: Data for the Effect of BM-23 (compound 17) on Kinase Activity**

| BM-23 (Log [nM]) | AuroraA Activity Luminescence | | | % Activity | | |
|---|---|---|---|---|---|---|
| | Set1 | Set2 | Set3 | Set1 | Set2 | Set3 |
| No Compound | 4863 | 4877 | 5271 | 97 | 97 | 105 |
| 0 | 4998 | 4727 | 4671 | 100 | 94 | 93 |
| 1.0 | 4852 | 4880 | 4833 | 97 | 97 | 97 |
| 1.5 | 5131 | 4948 | 5289 | 103 | 99 | 106 |
| 2.0 | 4851 | 5011 | 4906 | 97 | 100 | 98 |
| 2.5 | 5093 | 4820 | 4740 | 102 | 96 | 95 |
| 3.0 | 3856 | 4095 | 3994 | 77 | 81 | 79 |
| 3.5 | 3297 | 3208 | 3170 | 65 | 63 | 63 |
| 4.0 | 2374 | 2142 | 2242 | 46 | 42 | 44 |
| 4.5 | 2130 | 2031 | 2019 | 41 | 39 | 39 |
| 5.0 | 1738 | 1784 | 1649 | 34 | 34 | 32 |
| Background | 93 | 95 | 89 | | | |

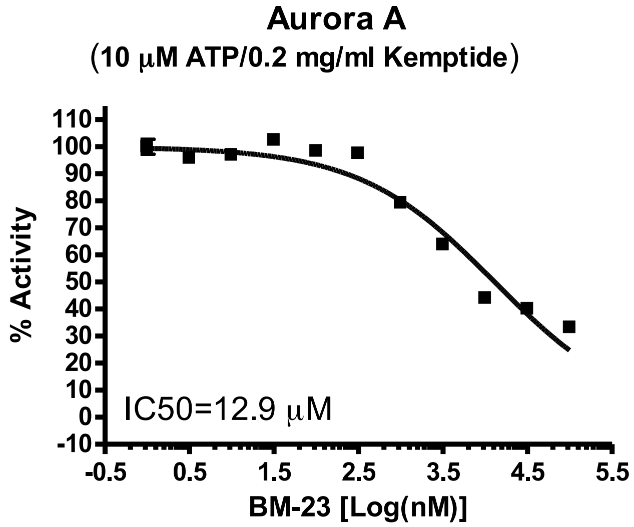

**Table S37: Data for the Effect of SZ-34 (compound 18) on Kinase Activity**

| SZ-34 (Log [nM]) | Aurora A Luminescence | | | % Activity | | |
|---|---|---|---|---|---|---|
| | Set1 | Set2 | Set3 | Set1 | Set2 | Set3 |
| No Compound | 6175 | 5774 | 5749 | 105 | 98 | 97 |
| 0 | 6502 | 5506 | 6122 | 110 | 93 | 104 |
| 1.0 | 6071 | 5505 | 5958 | 103 | 93 | 101 |
| 1.5 | 6174 | 5661 | 6189 | 105 | 96 | 105 |
| 2.0 | 6071 | 5522 | 5887 | 103 | 93 | 100 |
| 2.5 | 5338 | 5074 | 5472 | 90 | 86 | 93 |
| 3.0 | 4583 | 4351 | 5029 | 77 | 73 | 85 |
| 3.5 | 3037 | 2695 | 2850 | 51 | 45 | 47 |
| 4.0 | 1970 | 1823 | 1902 | 32 | 30 | 31 |
| 4.5 | 1784 | 1589 | 1644 | 29 | 26 | 26 |
| 5.0 | 1824 | 1589 | 1623 | 30 | 26 | 26 |
| Background | 104 | 109 | 127 | | | |

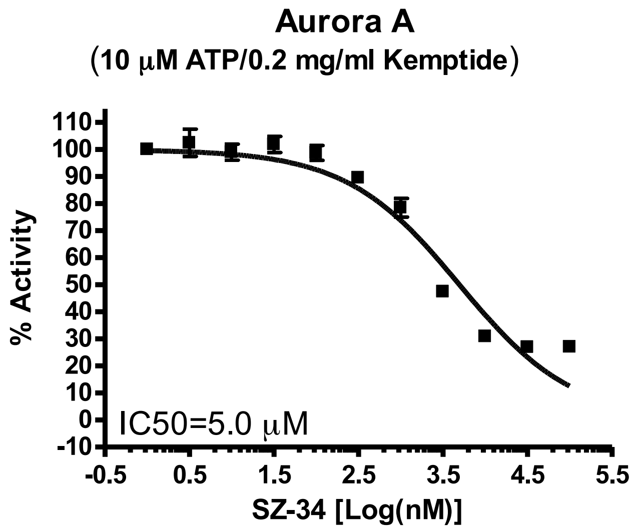

**Table S38: Data for the Effect of Staurosporine on AURKA Activity**

| Staurosporine (Log [nM]) | AuroraA Activity Luminescence | | | % Activity | | |
|---|---|---|---|---|---|---|
| | Set1 | Set2 | Set3 | Set1 | Set2 | Set3 |
| No Compound | 4725 | 4805 | 5039 | 97 | 99 | 104 |
| -1.0 | 4868 | 4529 | 4954 | 100 | 93 | 102 |
| -0.5 | 4781 | 4837 | 4938 | 98 | 100 | 102 |
| 0 | 5302 | 4980 | 5271 | 109 | 103 | 109 |
| 1.0 | 4961 | 4893 | 4971 | 102 | 101 | 102 |
| 1.5 | 3565 | 3380 | 4147 | 73 | 69 | 85 |
| 2.0 | 484 | 544 | 472 | 8 | 9 | 8 |
| 2.5 | 232 | 239 | 224 | 3 | 3 | 3 |
| 3.0 | 178 | 183 | 227 | 2 | 2 | 3 |
| 3.5 | 194 | 185 | 204 | 2 | 2 | 2 |
| 4.0 | 179 | 176 | 178 | 2 | 2 | 2 |
| Background | 102 | 90 | 96 | | | |

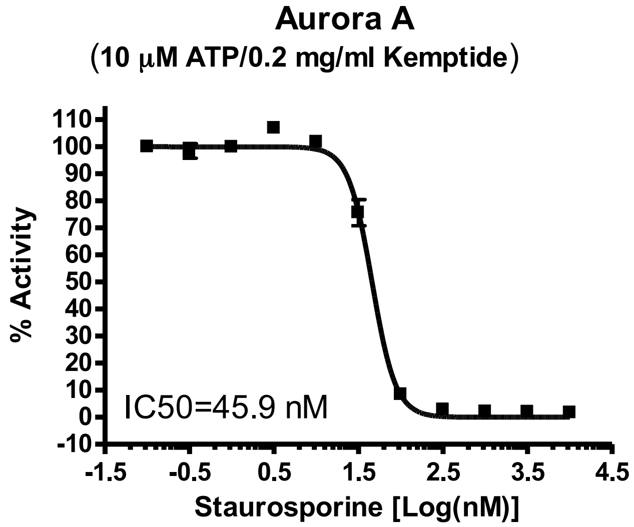

**Binding Affinities for EGFR, AURKA and AURKB**

The assay was performed externally at DiscoverX Corporation using a competition binding assay that quantitatively measures the ability of a compound to compete with an immobilized, active-site directed ligand for target kinases AURKA, AURKB and EGFR.[23] The assay is performed by combining three components: DNA-tagged kinase; immobilized ligand; and a test compound. The ability of the test compound to compete with the immobilized ligand was measured via quantitative PCR of the DNA tag. An 11-point 3-fold serial dilution of each test compound was prepared in 100% DMSO at 100x final test concentration and subsequently diluted to 1x in the assay (final DMSO concentration = 1%). Compound Kds were determined using a compound top concentration = 30,000 nM. If the initial Kd determined was < 0.5 nM (the lowest concentration tested), the measurement was repeated with a serial dilution starting at a lower top concentration. Binding constants (Kds) were calculated with a standard dose-response curve using the Hill equation.

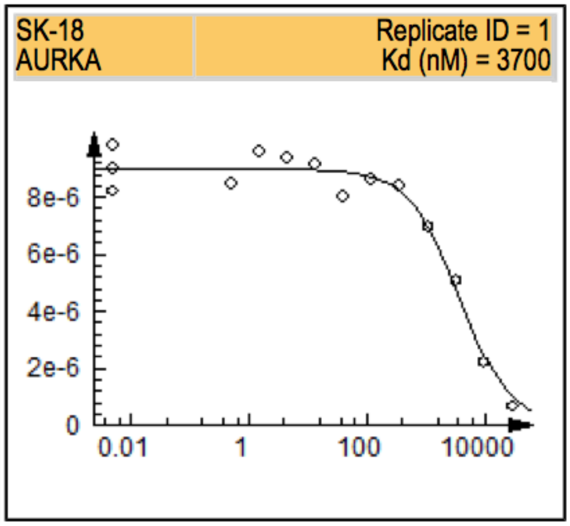


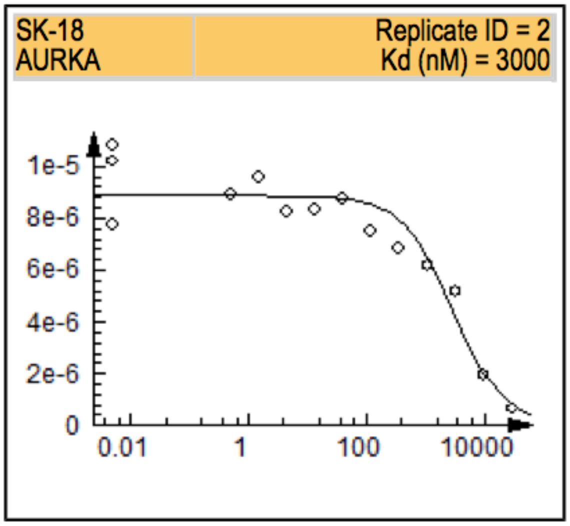


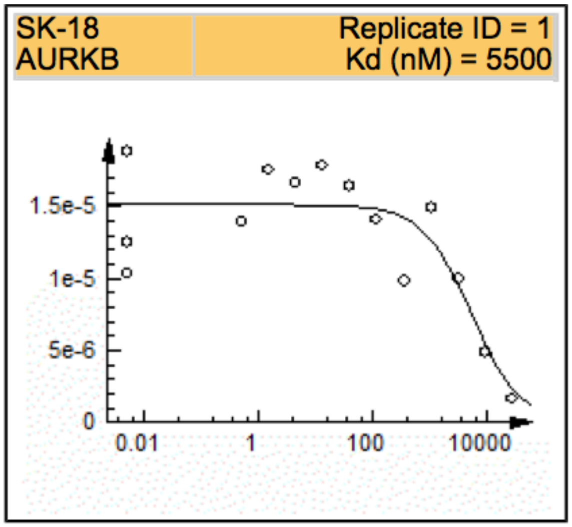


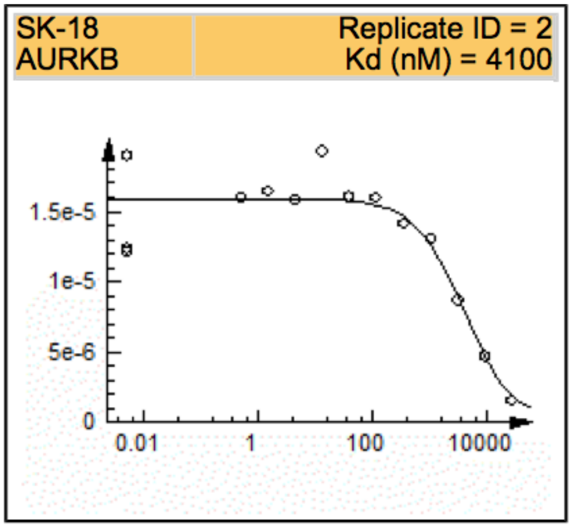

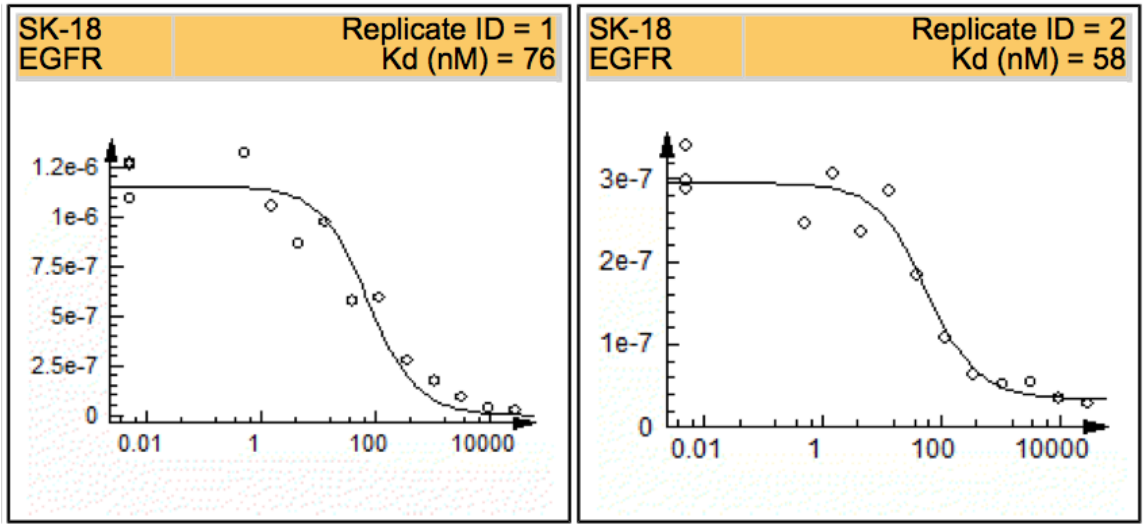
SK-18
EGFR
Replicate ID = 1
Kd (nM) = 76
1.2e-6
1e-6
7.5e-7
5e-7
2.5e-7
0
0.01
1
100
10000
SK-18
EGFR
Replicate ID = 2
Kd (nM) = 58
3e-7
2e-7
1e-7
0
0.01
1
100
10000
